# Intra-patient Inter-metastatic Genetic Heterogeneity in Colorectal Cancer as a Key Determinant of Survival after Curative Liver Resection

**DOI:** 10.1371/journal.pgen.1006225

**Published:** 2016-07-29

**Authors:** Anita Sveen, Inger Marie Løes, Sharmini Alagaratnam, Gro Nilsen, Maren Høland, Ole Christian Lingjærde, Halfdan Sorbye, Kaja Christine Graue Berg, Arild Horn, Jon-Helge Angelsen, Stian Knappskog, Per Eystein Lønning, Ragnhild A. Lothe

**Affiliations:** 1 Department of Molecular Oncology, Institute for Cancer Research, Oslo University Hospital, Oslo, Norway; 2 K.G. Jebsen Colorectal Cancer Research Centre, Oslo University Hospital, Oslo, Norway; 3 Centre for Cancer Biomedicine, Faculty of Medicine, University of Oslo, Oslo, Norway; 4 Department of Clinical Science, University of Bergen, Bergen, Norway; 5 Department of Oncology, Haukeland University Hospital, Bergen, Norway; 6 Department of Computer Science, University of Oslo, Oslo, Norway; 7 Department of Digestive Surgery, Haukeland University Hospital, Bergen, Norway; 8 Department of Clinical Medicine, University of Bergen, Bergen, Norway; Arizona State University & University of California San Francisco, UNITED STATES

## Abstract

Chromosomal instability is a well-defined hallmark of tumor aggressiveness and metastatic progression in colorectal cancer. The magnitude of genetic heterogeneity among distinct liver metastases from the same patient at the copy number level, as well as its relationship with chemotherapy exposure and patient outcome, remains unknown. We performed high-resolution DNA copy number analyses of 134 liver metastatic deposits from 45 colorectal cancer patients to assess: (i) intra-patient inter-metastatic genetic heterogeneity using a heterogeneity score based on pair-wise genetic distances among tumor deposits; and (ii) genomic complexity, defined as the proportion of the genome harboring aberrant DNA copy numbers. Results were analyzed in relation to the patients’ clinical course; previous chemotherapy exposure and outcome after surgical resection of liver metastases. We observed substantial variation in the level of intra-patient inter-metastatic heterogeneity. Heterogeneity was not associated with the number of metastatic lesions or their genomic complexity. In metachronous disease, heterogeneity was higher in patients previously exposed to chemotherapy. Importantly, intra-patient inter-metastatic heterogeneity was a strong prognostic determinant, stronger than known clinicopathological prognostic parameters. Patients with a low level of heterogeneity (below the median level) had a three-year progression-free and overall survival rate of 23% and 66% respectively, *versus* 5% and 18% for patients with a high level (hazard ratio0.4, 95% confidence interval 0.2–0.8, P = 0.01; and hazard ratio0.3,95% confidence interval 0.1–0.7, P = 0.007). A low patient-wise level of genomic complexity (below 25%) was also a favorable prognostic factor; however, the prognostic association of intra-patient heterogeneity was independent of genomic complexity in multivariable analyses. In conclusion, intra-patient inter-metastatic genetic heterogeneity is a pronounced feature of metastatic colorectal cancer, and the strong prognostic association reinforces its clinical relevance and places it as a key feature to be explored in future patient cohorts.

## Author Summary

Liver metastasis from colorectal cancer confers a poor patient prognosis and is often manifested as multiple independent lesions. Clonal evolution is an important biological process in metastatic development, but the magnitude and clinical relevance of genetic heterogeneity among metastatic deposits in individual patients remains unknown. We describe for the first time a large variation among patients in the level of inter-metastatic heterogeneity at the DNA copy number level in liver metastases from colorectal cancer. We found this heterogeneity to be a biological feature independent both of the number of metastases per patient and of the extent of copy number aberrations (genomic complexity)of the metastases. Previous chemotherapy exposure was associated with a higher patient-wise level of heterogeneity in subsequent metastases. Importantly, we discovered inter-metastatic heterogeneity to be a key determinant of patient survival and a stronger prognostic factor than known clinicopathological parameters of metastatic colorectal cancer. This study reinforces the clinical relevance of tumor heterogeneity and we identify inter-metastatic heterogeneity as an important feature to explore in future cohorts of patients with metastatic colorectal cancer.

## Introduction

Colorectal cancer (CRC) is the third most common type of cancer world-wide, responsible for almost 700,000 deaths each year[1]. Liver metastases are a major cause of death from CRC, and approximately 20% of patients present with synchronous liver metastases[2], while another 40% develop metachronous metastases after resection of the primary tumor[2–4]. Colorectal tumors are frequently affected by chromosomal instability, a type of genomic instability causing aneuploidy, characterized by numerous DNA copy number changes and structural aberrations[5–7]. Chromosomal instability is associated both with a poor patient prognosis[8]and multidrug resistance [9], and has recently been implicated as a requirement for metastatic progression in models of CRC [10]. Consequently, most liver metastases have chromosomal instability.

Specific DNA copy number aberrations in primary tumors from patients with metastatic disease have been associated both with metastatic progression[11–13] and patient prognosis after chemotherapy[14]. However, despite substantial genetic tumor heterogeneity and branched evolutionary patterns described in metastatic CRC, most DNA copy number aberrations, and in particular aberrations affecting well-known cancer-critical genes, were recently found to be shared among multiple samples from paired primary and metastatic lesions [15]. Furthermore, although a few aberrations specific to metastatic deposits have been described, the DNA copy number profiles of liver metastases are generally similar to those of primary CRCs [13,16–20]. Thus, our understanding of genetic factors regulating the metastatic process remains poor[20,21].

Emerging evidence indicates intra-tumor heterogeneity and clonal evolution to play a pivotal role in cancer progression[21–24]. Genetic alterations between primary tumors and their metastatic deposits[15,18,19,25–31] may have several causes, including spontaneous evolution of aggressive subclones or clonal selection due to adjuvant chemotherapy, but also the mutagenic effect of chemotherapeutic drugs[32,33]. In CRC, platinum-based chemotherapy is frequently given in the adjuvant setting and platinum drug exposure has been associated with an increased incidence of *KRAS* mutations in subsequent liver metastases[34]. Although chemotherapy exposure many influence the biology of liver metastases, the clinical implications remain largely unknown.

While it is generally assumed that a limited number of primary tumor cells possess metastatic propensity[35], we do not know whether each individual primary tumor may harbor several different cell clones able to metastasize. However, multiple metastases often develop synchronously years after curative-intended treatment for the primary tumor. Taken together, these factors illuminate the need not only to study somatic changes in tumors over time, but also to explore potential metastatic heterogeneity. For most cancer forms, the feasibility of studying genetic alterations across multiple distinct metastatic deposits from an individual tumor is limited. Over the last decades, liver resection for metastatic CRC has been implemented as routine therapy, leading to a 5-year survival rate of 35–40%among eligible patients [36]. Unfortunately, disease will recur in at least 70% of resected patients[37]. Several clinicopathological parameters and *BRAF* mutations are known prognostic determinants of recurrence and survival after liver surgery; however, their clinical use is limited[36,38].

Resected liver specimens offer a unique setting to explore genetic alterations across multiple metastatic deposits collected from the same recipient organ and, accordingly, CRC represents an attractive model disease for analysis of metastatic heterogeneity. In addition, liver resection offers an opportunity for examination of prognostic factors for metastatic CRC, as well as to investigate whether biological heterogeneity by itself may be related to patient outcome. Here, we analyzed for the first time the magnitude and clinical relevance of intra-patient inter-metastatic heterogeneity at a high resolution DNA copy number level in patients with resectable liver metastases. We identified a large variation among patients in the level of heterogeneity, and showed that this variation was unrelated to the number of metastatic deposits and the average genomic complexity of the tumors. High inter-metastatic heterogeneity was a key determinant of poor patient outcome after partial liver resection, and a stronger prognostic factor than known clinicopathological factors, reinforcing the importance and clinical relevance of this biological process.

## Results

### Intra-patient genetic heterogeneity in liver metastases

**Fig 1:**
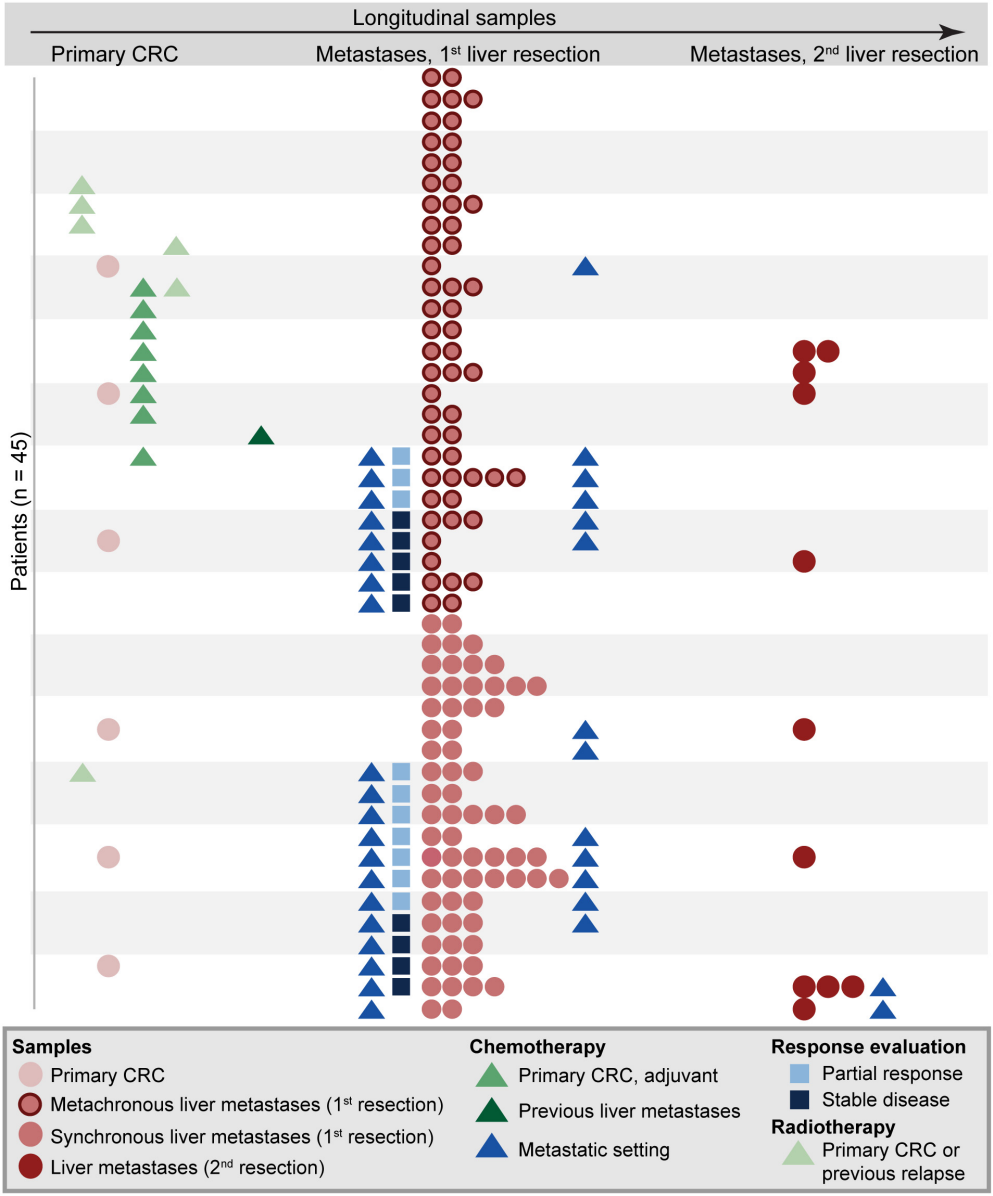
Overview of tumor samples analyzed and treatment course for each patient. A total of 140 samples from primary CRCs (n = 6) and liver metastases (n = 134; 123 and 11 from first and second liver resections respectively) from 45 patients were included. Each row represents one patient. The patients are ordered according to metachronous (top) or synchronous (bottom) presentation of liver metastases, and according to treatment with chemotherapy. On the horizontal axis, a timeline is included to indicate the order of events. CRC, colorectal cancer.

**Fig 2:**
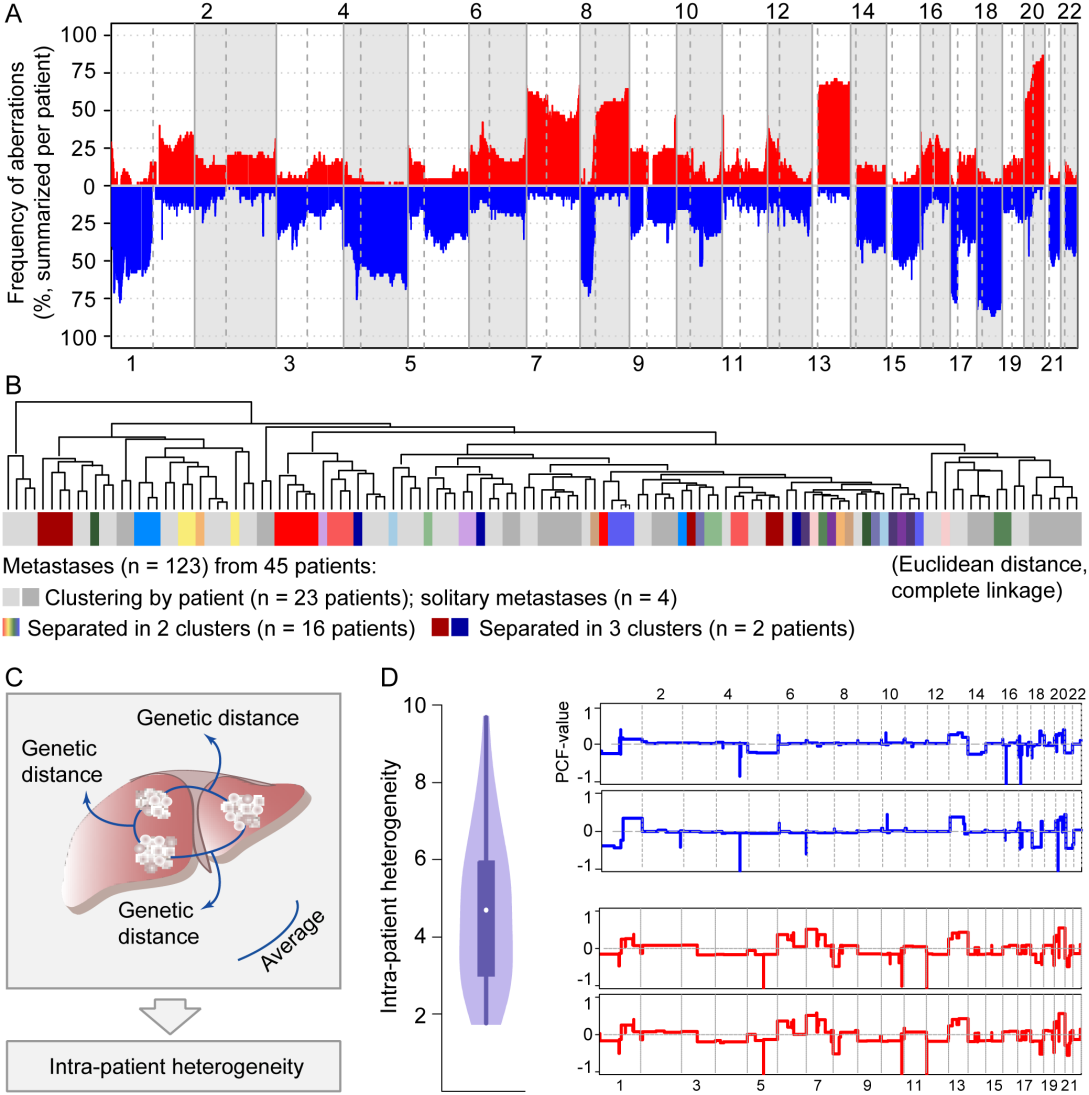
Variation in intra-patient genetic heterogeneity in liver metastases. (A) Summarized patient-wise, the DNA copy number profiles of liver metastases (n = 134 from 45 patients) show high-frequency gains (vertical axis; red) on chromosome arms 7p,7q, 8q, 13q, and 20q, and losses (blue) on 1p, 4p, 4q, 8p, 17p, and 18q. In this plot, chromosome numbers are indicated on the horizontal axes, and chromosomes and chromosome arms are separated by vertical lines. (B) Hierarchical clustering of metastatic deposits collected at first liver resection (n = 123 from 45 patients)based on DNA copy numbers. In 23 patients, the metastatic deposits clustered in a patient-wise manner (different tones of grey distinguish among metastases from individual patients plotted adjacent to each other). Four patients had solitary metastases. In 18 patients, the metastatic deposits were separated in multiple clusters (individual color for each patient), indicating intra-patient heterogeneity. Clustering was done by complete linkage, based on Euclidean distance metrics. (C)Intra-patient inter-metastatic genetic heterogeneity was measured as the average pair-wise Euclidean distance of genome-wide copy numbers among all metastases from each patient (illustrated for a patient with three liver metastases). (D) Violin plot (left) showing the distribution of intra-patient inter-metastatic genetic heterogeneity among the 41 patients with multiple metastatic deposits collected at first liver resection (range 1.7 to 9.7; median 4.7). The right panel shows representative individual copy number segmentation profiles of two metastases from a patient with a level of heterogeneity above (5.0; blue) and below (2.6; red) the median level.

To assess genetic heterogeneity in metastatic CRC, genome-wide copy number profiles of a total of 134 liver metastases obtained by partial liver resection of 45 patients with metastatic CRC were analyzed (Table 1 and Fig 1). The median and mean number of metastatic deposits collected at first liver resection was 2 and 2.7 respectively (range 1–7), with 41 of 45 patients harboring at least two deposits. The frequency of DNA copy number aberrations in the liver metastases (Fig 2A) were summarized patient-wise (each patient was considered to have an aberration if found in at least one metastatic deposit), and the liver metastases showed similar aberrations and aberration frequencies to primary CRCs (Results in S1 Text and Fig A in S2 Text).

**Table 1:** Patient characteristics and treatment (n = 45).

Age at diagnosis, mean (range)	63 (41–83)
Sex (male, female)	25, 20
Years of follow-up, mean (range)	2.4 (0–4.8)
Primary tumor site (colon, rectum, multiple)	35, 8, 2
Nodal status of primary cancer (pN0, pN+)	17, 28
Synchronous or metachronous liver metastases^a^	20, 25
Number of liver metastases on imaging at diagnosis^b^:	
-Mean (range)	3 (1–8)
-Patients with multiple metastases	35
-Patients with single metastasis	10
Number of sampled and analyzed liver metastases (first liver resection):	
-Mean (range)	3 (1–7)
-Patients with multiple metastases	41
-Patients with single metastasis	4
Size (cm) of largest lesion at start of treatment, mean (range)	4 (1–11)
Patients with largest metastasis >5 cm	14
Patients with extrahepatic disease	4
Adjuvant chemotherapy after resection of primary tumor:	
-5FU, folinic acid	2
-5FU, oxaliplatin, folinic acid	6
Previous radiotherapy^c^:	
- with low-dose chemotherapy	4
- without chemotherapy	2
Chemotherapy prior to liver resection^d^	20
Best response on chemotherapy (partial response, stable disease)^e^	10, 9
CEA (<30, >30, missing)	18, 13, 14
MSI-status (MSI, MSS, non-determined)	1, 42, 2
WHO performance status (0, I, II)	36, 8, 1

^a^Metastases were considered to be metachronous if diagnosed ≥three months after diagnosis of the primary tumor;

^b^The numbers of metastases available for sampling during surgery may be higher or lower, due to detection limits, time and treatment;

^c^Four patients were treated for the primary tumor and two patients for previous relapse;

^d^Three or more courses of treatment (except two patients who had to stop treatment after one and two cycles respectively, due to side effects);

^e^RECIST 1.1. Not calculated for the patient receiving only one treatment course.

5FU, fluorouracil; CEA, Carcinoembryonic Antigen; MSI, microsatellite instability; MSS, microsatellite stable; RECIST, Response Evaluation Criteria in Solid Tumors.

To explore the extent of intra-patient inter-metastatic heterogeneity, hierarchical clustering was performed on all 123 metastatic deposits collected at first liver resection, based on genome-wide copy number estimates (for atomic segments with variance higher than 0.03 across all samples). This revealed two distinct groups of patients. In the first group of 23 patients (56% of the 41 patients with multiple liver deposits), all metastatic deposits clustered in a patient-wise manner (Fig 2B), with a probability value for each cluster higher than 95% for 19of the patients, assessed by bootstrap resampling. By contrast, in the second group of 18 patients (44% of the 41 patients with multiple liver deposits), there was larger intra-patient variation, with one or more metastatic deposits revealing a DNA copy number profile more similar to unrelated metastases (the probabilities of the separating clusters were higher than 90% for eight of these patients). This indicated variation among the patients in the level of inter-metastatic heterogeneity.

To quantify the level of heterogeneity per patient, we calculated a patient-wise heterogeneity score similar to the genetic divergence measure previously used to analyze clonal diversity in Barrett’s esophagus[39]. Intra-patient inter-metastatic genetic heterogeneity was calculated as the average Euclidean distance of genome-wide copy number estimates (for copy number segments with variance across all samples exceeding 0.03) for all possible pair-wise comparisons of liver metastases from the same patient (for the 41 patients with multiple metastases from the first liver resection; illustrated in Fig 2C). The patient-wise level of heterogeneity spanned a ratio of five, ranging from 1.7 to 9.7, with a median of 4.7 (Fig 2D), further demonstrating the variation among the patients in the level of inter-metastatic heterogeneity. For comparison with this Euclidean distance-based method, intra-patient inter-metastatic heterogeneity was also calculated as the average pair-wise proportion of the genome of the metastases with different copy numbers (summarized from segments with differences in copy number estimates larger than 0.1). There was a strong internal consistency between the values obtained from the two calculation methods(Pearson correlation 0.8, P < 0.0001; Fig B in S2 Text), underlining the robustness of our method.

**Fig 3:**
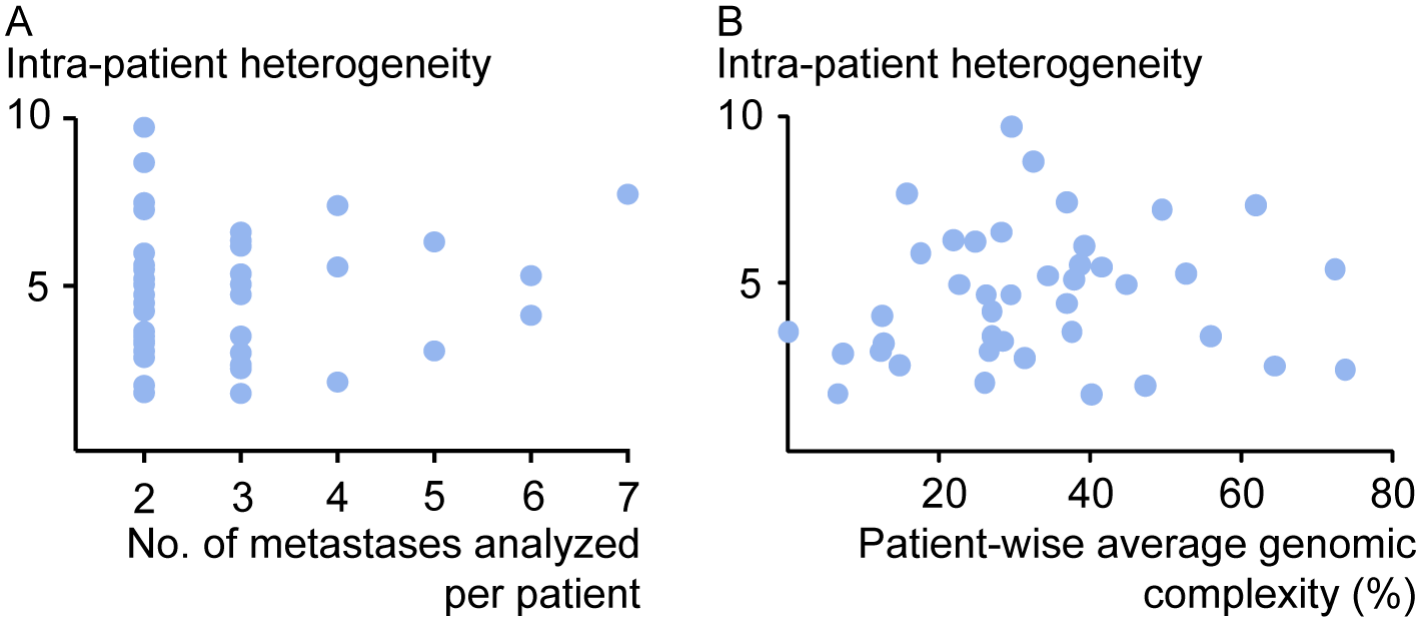
Intra-patient inter-metastatic genetic heterogeneity is independent of the number and genomic complexity of liver metastases. For the 41 patients with multiple metastases from the first liver resection (blue dots), the level of intra-patient inter-metastatic genetic heterogeneity was not associated with either (A) the number of metastases analyzed per patient (Pearson correlation 0.1, P = 0.5), or (B) the patient-wise average genomic complexity across the metastases (calculated from the per cent of base pairs with aberrant copy numbers per metastasis; Pearson correlation 0.1, P = 0.5).

Importantly, the patient-wise level of heterogeneity was not associated with the number of metastatic deposits analyzed in the individual patient (Pearson correlation 0.1, P = 0.5; Fig 3A). Also, the association with the size of the largest liver metastatic deposit per patient was weak (Pearson correlation 0.3, P = 0.06; Fig B in S2 Text). Furthermore, the cancer cell fraction of the samples from the individual metastatic deposits was estimated based on the DNA copy number data using ASCAT [40], and was found to range from 26% to 95% (median 64%; ASCAT was unable to estimate the cancer cell fraction of six samples). There was only a weak association between the intra-patient genetic heterogeneity and patient-wise diversity in the cancer cell fraction of the metastatic samples (calculated as the absolute difference in the cancer cell fraction of all possible pair-wise comparisons of metastases from each patient; Pearson correlation 0.4, P = 0.02; Fig B in S2 Text), indicating that the cancer cell fraction alone cannot explain the genetic heterogeneity identified.

### Genomic complexity in liver metastases

As a measure of chromosomal instability, the genomic complexity of each metastatic deposit was calculated as the proportion of the genome (per cent of base pairs) with aberrant copy numbers. Twenty-two (16%) of the 134 metastatic deposits had few DNA copy number aberrations and a level of genomic complexity below 10%. Considering the largely mutually exclusive relationship between chromosomal instability and microsatellite instability (MSI), MSI-status was determined for one metastatic deposit per patient. Consistent with a low prevalence of MSI in metastatic CRC[41], only one of these deposits was found to have MSI (2%; Table 1). As expected, this sample, together with the second metastatic deposit from the same patient, showed the lowest levels of genomic complexity (lower than 0.01%). There was no clear association between the level of genomic complexity and cancer cell fraction of the samples(Fig C in S2 Text). Furthermore, there was no difference in the estimated cancer cell fraction between samples with a level of genomic complexity below and above 10% (P = 0.5; independent samples t-test).

As a patient-wise measure of genomic complexity, the average complexity across all metastatic deposits collected at first liver resection of each patient was calculated and found to range from 0 to 74% (median 28%; Fig C in S2 Text). Consistent with a recent pan-cancer analysis of primary tumors[42], we found that a level of genomic complexity below 25% was associated with a favorable patient outcome also in metastatic CRC. Patients with a level of genomic complexity in liver metastases lower than this threshold (n = 16) had a three-year progression-free survival (PFS)rate of 28%, significantly higher than the 10% PFS rate in patients with a genomic complexity above the 25% level (n = 29; hazard ratio, HR, calculated by Cox’s regression 0.4 [95% confidence interval 0.2–0.9]; P = 0.02,calculated from Wald’s test of predictive potential). Furthermore, the three-year overall survival (OS) rate was 77% for patients with a level of genomic complexity below 25%, significantly higher than the 29% OS rate in patients with the higher levels of genomic complexity (HR = 0.2 [0.06–0.7], P = 0.01; Fig C in S2 Text).

Interestingly, among patients (n = 41) with two or more metastatic deposits at first liver resection, no association between the patient-wise average genomic complexity and intra-patient genetic heterogeneity was recorded (Pearson correlation 0.1, P = 0.5; Fig 3B), indicating that a high level of inter-metastatic genetic heterogeneity may occur both among metastases with a large and small proportion of the genome affected by DNA copy number aberrations. Thirteen (32%) of the 41 patients had at least one metastatic deposit with a level of genomic complexity lower than 10%. Even when excluding these 13 patients, no association between intra-patient genetic heterogeneity and genomic complexity was recorded (Pearson correlation 0.2, P = 0.4).

### Mutations of *TP53* in liver metastases

Considering that *TP53* mutations have been found to increase the tolerance of tumor cells to DNA copy number aberrations [43], all metastatic deposits were analyzed for *TP53* mutations. Sixty-two per cent (28 of 45) of the patients had a mutation in at least one metastatic deposit collected at first or second liver resection, with five patients (11%) displaying heterogeneity (both wild-type and mutated metastases). *TP53* mutations were found to be associated with a high patient-wise level of genomic complexity in liver metastases, reinforcing the link between chromosomal instability and *TP53* mutations. Patients (n = 23) with *TP53* mutations in all metastatic deposits (from both first and second liver resection), had significantly higher genomic complexity in their metastatic deposits than patients (n = 17) for whom all metastatic deposits harbored wild-type *TP53*. The median patient-wise genomic complexity among patients with and without *TP53* mutations was 37% and 23% respectively (P = 0.03,independent samples t-test; Fig D in S2 Text). The genomic regions with largest differences in copy number aberration frequencies between metastatic deposits with and without *TP53* mutations are listed in Table A in S3 Text.*TP53* mutations were not significantly associated with patient survival. Furthermore, there was no difference in intra-patient inter-metastatic heterogeneity at the DNA copy number level among patients with *TP53* wild-type, mutated, or heterogeneously mutated metastatic deposits (Fig E in S2 Text).

### Influence of chemotherapy exposure on intra-patient inter-metastatic genetic heterogeneity

**Fig 4:**
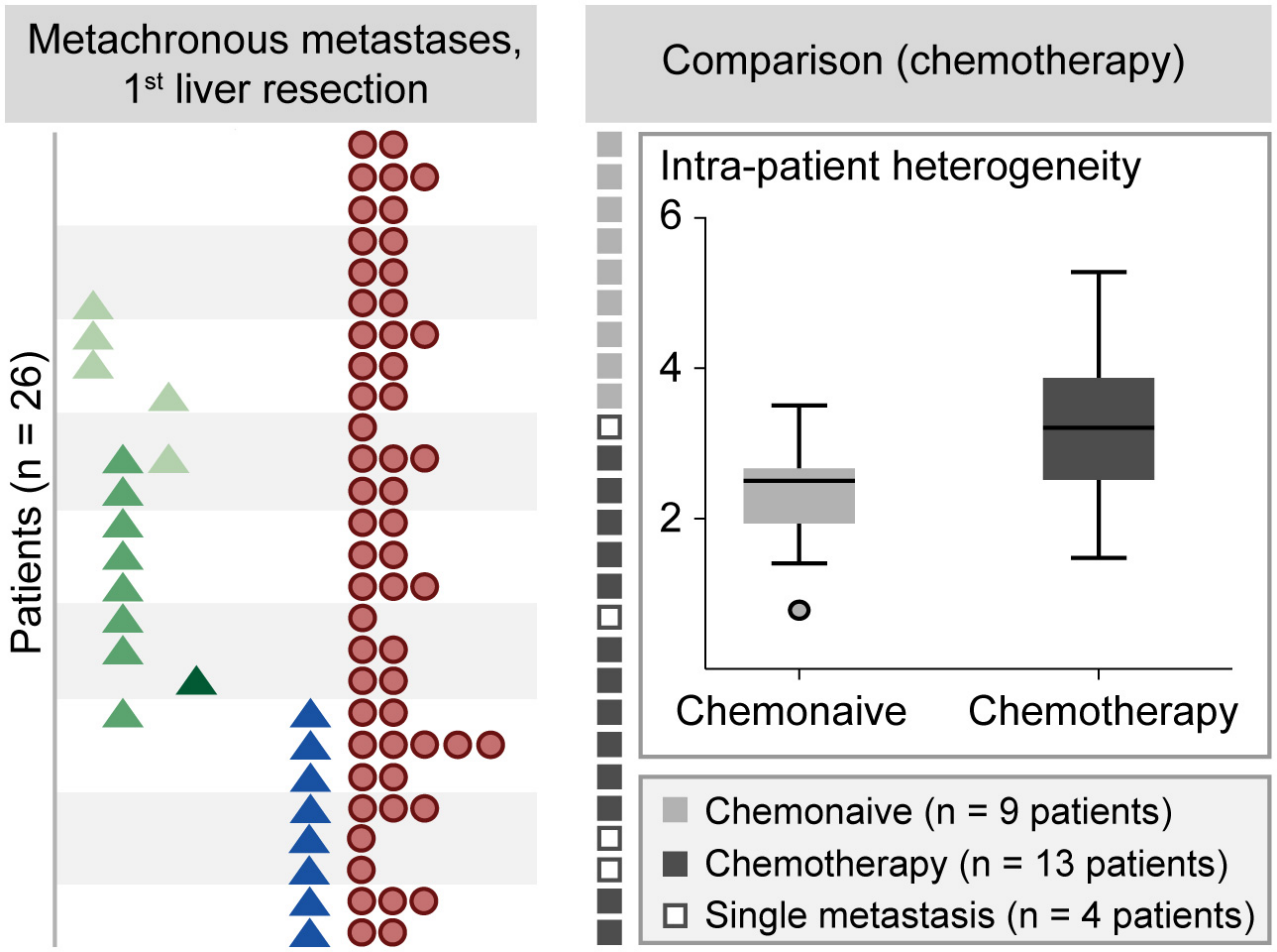
High intra-patient inter-metastatic genetic heterogeneity after chemotherapy exposure. Twenty-two of the 26 patients (vertically in the left panel) with metachronous metastases had two or more liver deposits (horizontally in the left panel) at first liver resection (the symbols are the same as described in Fig 1). The right panel shows the intra-patient inter-metastatic genetic heterogeneity in chemonaïve patients (n = 9; indicated by light grey boxes in the left part of the right panel) *versus* patients treated with chemotherapy (n = 13; indicated by dark grey boxes), respectively, demonstrating higher intra-patient inter-metastatic heterogeneity after chemotherapy exposure (P = 0.03 by independent samples t-test; however, not statistically significant after correcting for multiple testing).

We observed no difference in heterogeneity between patients harboring synchronous (n = 19 patients) and metachronous (n = 22 patients) metastases (independent samples t-test, P = 0.3; Fig F in S2 Text). Analyzing all patients harboring metachronous and synchronous metastases together, no difference with respect to heterogeneity was recorded between patients exposed to chemotherapy(FOLFOX [44] or Nordic FLOX [45]; n = 25) and non-exposed patients (n = 16). Similarly, among patients with synchronous metastases, no difference in heterogeneity between chemonaïve (n = 7) and treated (n = 12) patients was observed (Fig F in S2 Text). By contrast, for patients with metachronous metastases, heterogeneity was higher among patients previously exposed to chemotherapy (n = 13) than among chemonaïve patients (n = 9; P = 0.03 by independent samples t-test; Fig 4), although this difference was not statistically significant after correcting for multiple testing (false discovery rate higher than 0.05). There was no difference in heterogeneity between patients treated with adjuvant chemotherapy for the primary CRC and chemotherapy just prior to liver resection (P = 0.9). Among patients receiving chemotherapy prior to liver resection, no difference in heterogeneity related to treatment response (stable disease or partial response), as evaluated according to the RECIST criteria [46], was recorded (Results in S1 Text and Fig G in S2 Text). The results were not influenced by previous radiotherapy or concomitant chemoradiotherapy (Figs F and H in S2 Text).

The regions with the highest increase in intra-patient DNA copy number variation after exposure to chemotherapy were located on chromosome arms 8q, 9q, 10q, 19p, and 20p (Results in S1 Text and Table B in S3 Text). In particular, a region on 20p, partly encoding the gene *MACROD2*, showed a high frequency of both gains and losses after chemotherapy exposure (Fig I in S2 Text).

To investigate longitudinal variation, intra-patient inter-metastatic heterogeneity was calculated as the average Euclidean distance of all possible pair-wise comparisons between metastatic deposits collected at the first and second liver resection of eight patients. Although the number of patients available for this comparison was small, exposure to chemotherapy was associated with a high level of intra-patient heterogeneity also in this setting. We observed higher longitudinal heterogeneity between metastases from the two resections in patients who received post-operative chemotherapy after resection for their first metastases (n = 2), compared to patients who had not received chemotherapy in this setting (n = 6; Fig J in S2 Text).

No difference in the patient-wise genomic complexity between patients exposed to chemotherapy and chemonaïve patients, nor in patients with synchronous compared to metachronous metastases was recorded (both P > 0.3; independent samples t-test). Among patients receiving chemotherapy prior to liver resection, no difference in genomic complexity related to treatment response (stable disease or partial response) was recorded (Fig G in S2 Text).

### Influence of intra-patient inter-metastatic genetic heterogeneity on patient outcome after liver resection

**Fig 5:**
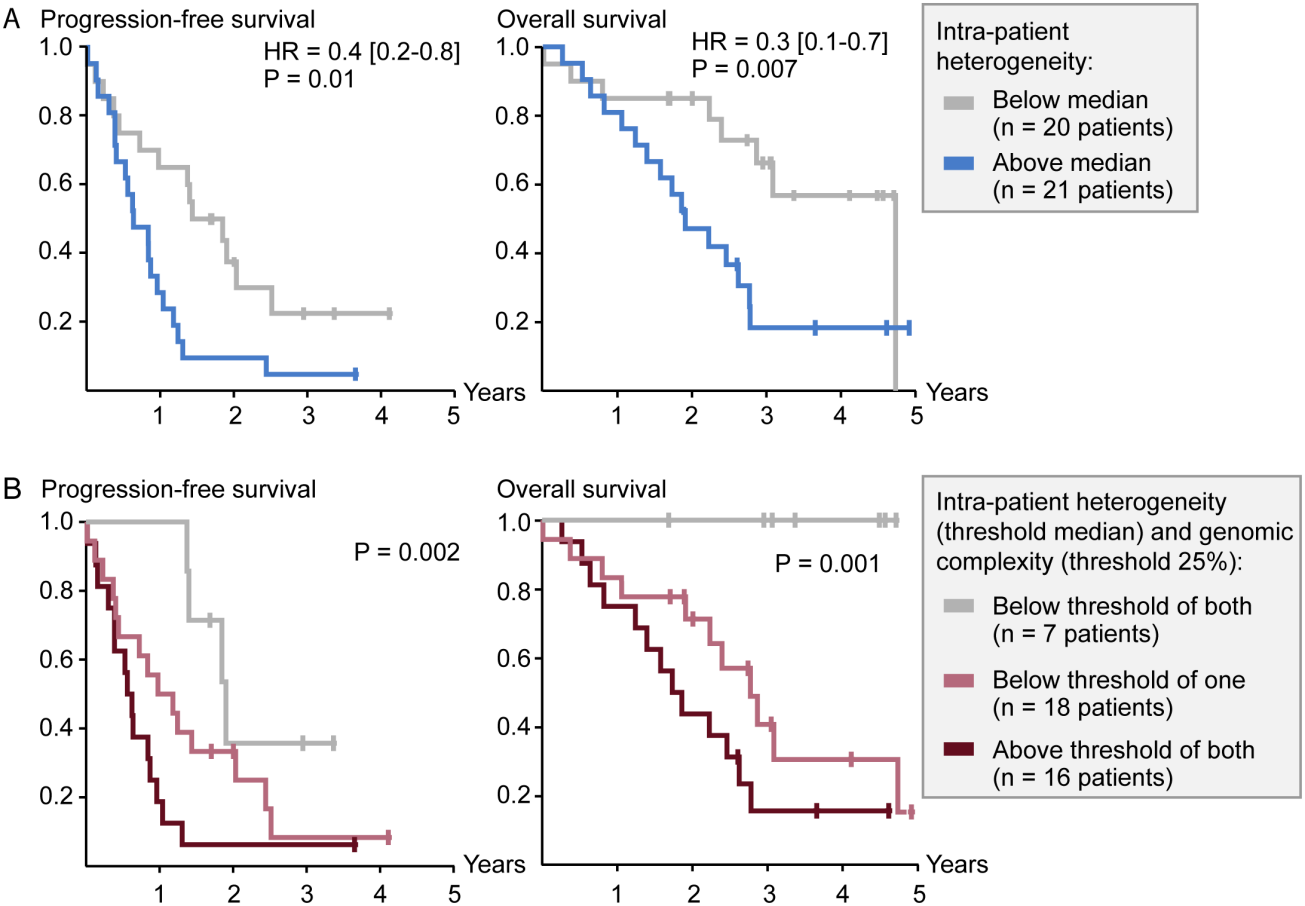
Low intra-patient inter-metastatic genetic heterogeneity and genomic complexity are associated with favorable patient outcome. (A) A low level of intra-patient inter-metastatic genetic heterogeneity (below median) was strongly associated with a higher three-year progression-free and overall survival rate, measured from start of treatment for the metastases. Median progression-free survival was 17 and 8 months and the three-year overall survival rate was 66% and 18%for patients with heterogeneity below and above the median level respectively. Hazard ratios (HR) at three years of follow-up were calculated by Cox’s regression and P-values from Wald’s tests of predictive potential. (B) Patients with a low level of both intra-patient inter-metastatic heterogeneity (below median) and average genomic complexity (below 25%) had a significantly higher three-year progression-free (P = 0.002) and overall (P = 0.001) survival rate than patients with a low level of only one, or neither, of the two characteristics. Median progression-free survival was 23, 12 and 7 months for the three patient groups respectively, and the three-year overall survival rate was 100%, 41% and 16% respectively. P-values were calculated by the log-rank test of linear trend among the three patient groups.

Analyzing all the 41 patients with two or more metastatic deposits at first liver resection together, the three-year PFS and OS rates were 13% and 41% respectively. To assess the effect of genetic heterogeneity on patient survival, the patient-wise inter-metastatic heterogeneity score was analyzed as a continuous variable by Cox’s regression. A continuously increasing level of heterogeneity among patients was associated with a continuously decreasing three-year survival rate (P = 0.01 and 0.003 for PFS and OS respectively). Considering that hierarchical clustering revealed inter-metastatic heterogeneity in close to 50% of the patients, the patients were separated in two groups based on the median level of the patient-wise inter-metastatic heterogeneity score. Patients with a low (n = 20) and high (n = 21) level of heterogeneity had a median PFS after surgery of 17 and 8 months respectively, with a three-year PFS rate of 23% and 5%(HR = 0.4 [0.2–0.8], P = 0.01; Fig 5A). Furthermore, patients with a low level of heterogeneity had a significantly higher three-year OS rate than patients with heterogeneity above the median level (66%*versus* 18% respectively; HR = 0.3 [0.1–0.7], P = 0.007). The same prognostic association was seen when intra-patient inter-metastatic heterogeneity was calculated as the average pair-wise proportion of the genome of the metastases with different copy numbers (Fig B in S2 Text). Applying different threshold values for heterogeneity and separating the patients in three groups based on the 33^rd^ and 67^th^ percentiles of the heterogeneity score (below, between and above respectively), there was a significant linear trend between favorable patient survival and a low level of inter-metastatic heterogeneity (P = 0.008 and 0.006for three-year PFS and OS respectively, log-rank test of linear trend; Fig K in S2 Text).

The prognostic association of intra-patient inter-metastatic genetic heterogeneity was not determined by the metastatic deposits having few DNA copy number aberrations. Excluding the 13 patients with at least one metastatic deposit having a level of genomic complexity below 10% and analyzing the remaining 28 patients, the three-year HR for patients with a level of inter-metastatic heterogeneity below *versus* above the median was 0.4 [0.2–1.0] and 0.3[0.1–1.0] for PFS and OS respectively (P = 0.04 and 0.05 respectively). Considering that the intra-patient genetic heterogeneity and genomic complexity scores were independent of each other (Fig 3B), and that both were individually associated with patient survival, these genomic measures were explored in combination. Patients with a low level of both intra-patient heterogeneity (below median level) and genomic complexity (below 25%) constituted a group of patients with a particularly favorable survival rate. Separating the patients in three groups (patients with both, only one, and neither of the parameters below the threshold level), patients (n = 7) with a low level of both intra-patient heterogeneity and genomic complexity had a three-year OS rate of 100%, significantly higher than the 41% and 16% OS rates for patients with a low level of only one (n = 18), or neither (n = 16) of the characteristics respectively (P = 0.001 from log-rank test of linear trend; Fig 5B). The corresponding median PFS was 23, 12 and 7months for the three patient groups respectively (P = 0.002 from log-rank test of linear trend).

Known clinicopathological prognostic parameters were also explored for prognostic associations among the patients (n = 41)harboring multiple metastatic deposits at their first liver resection (Table 2). High patient age was a poor prognostic factor for three-year OS, while synchronous metastasis was a poor prognostic factor, compared with metachronous metastasis, for three-year PFS. The other clinicopathological parameters, including patient gender, nodal status of the primary cancer, the size of the largest liver metastasis and the number of radiologically diagnosed metastases were not prognostic for either PFS or OS. Notably, previous exposure to chemotherapy was not associated with patient survival. Multivariable analysis was performed for all parameters found to have prognostic associations in univariable analyses (P < 0.1, Table 2). For three-year OS, intra-patient inter-metastatic genetic heterogeneity was an independent prognostic determinant (multivariable HR = 0.2 [0.07–0.6], P = 0.003) in a multivariable model with patient age and the patient-wise genomic complexity (the results remained unchanged when including also synchronous *versus* metachronous metastases in the multivariable model). For three-year PFS, the prognostic association of intra-patient heterogeneity remained borderline significant (multivariable HR = 0.5 [0.2–1.1], P = 0.08) in a multivariable model with genomic complexity and synchronous *versus* metachronous metastases. Notably, in multivariable analyses including intra-patient heterogeneity and either genomic complexity or synchronous *versus* metachronous metastases separately, intra-patient heterogeneity was a significant independent prognostic determinant of PFS in both (multivariable HR = 0.4[0.2–0.8] and P = 0.02in both models).

**Table 2:** Intra-patient inter-metastatic genetic heterogeneity and patient survival.

	Three-year progression-free survival				Three-year overall survival			
	Univariable		Multivariable		Univariable		Multivariable	
	HR	P-value^b^	HR	P-value^b^	HR	P-value^b^	HR	P-value^b^
	[95% CI]^a^		[95% CI]^a^		[95% CI]^a^		[95% CI]^a^	
Intra-patient inter-metastatic genetic heterogeneity^c^ (below *vs*. above median)	0.4 [0.2–0.8]	0.01	0.5 [0.2–1.1]	0.08	0.3 [0.1–0.7]	0.007	0.2 [0.07–0.6]	0.003
Patient-wise average genomic complexity^d^ (below *vs*. above 25%)	0.5 [0.2–1.1]	0.07	0.4 [0.2–0.9]	0.03	0.3 [0.08–0.9]	0.03	0.3 [0.09–1.2]	0.09
*TP53* mutations (mutated *vs*. wild type)^e^	1.6 [0.8–3.3]	0.2	na	na	1.0 [0.4–2.5]	0.9	na	na
Patient age (continuous)	1.0 [0.99–1.1]	0.4	na	na	1.1 [1.0–1.1]	0.04	1.1 [1.02–1.1]	0.003
Patient gender (female *vs*. male)	1.1 [0.5–2.1]	0.9	na	na	0.9 [0.6–1.4]	0.7	na	na
Synchronous *vs*. metachronous metastases	2.0 [1.0–4.0]	0.05	2.5 [1.1–5.7]	0.02	1.3 [0.6–3.0]	0.5	na	na
Nodal status of primary CRC (lymph node positive *vs*. negative)	1.3 [0.6–2.7]	0.5	na	na	0.9 [0.3–2.1]	0.7	na	na
Size of largest liver metastasis (larger *vs*. smaller than five cm)	1.1 [0.6–2.3]	0.7	na	na	1.1 [0.5–2.8]	0.8	na	na
No. of liver metastases at diagnosis^f^ (multiple *vs*. single)	1.0 [0.4–2.6]	>0.9	na	na	2.0 [0.5–8.4]	0.4	na	na
Chemotherapy *vs*. no chemotherapy	1.2 [0.6–2.5]	0.6	na	na	0.7 [0.3–1.7]	0.5	na	na
Patient-wise diversity in cancer cell fraction^g^ (below *vs*. above median)	0.7 [0.3–1.4]	0.3	na	na	0.9 [0.4–2.1]	0.8	na	na

^a^Hazard ratios from Cox's regression;

^b^P-values from Wald's test of predictive potential;

^c^Calculated as the average Euclidean distance of genome-wide copy number estimates for all possible pair-wise comparisons of liver metastases from the same patient;

^d^Calculated from the per cent of base pairs of the metastases with aberrant copy numbers;

^e^Four patients with both mutated and wild type metastases from the first liver resection were excluded from the analyses;

^f^Six of the patients had a single liver metastasis on imaging at diagnosis;

^g^Calculated as the absolute difference in the cancer cell fraction (based on ASCAT estimates) of all possible pair-wise comparisons of metastases from each patient.

CI, confidence interval; na, not assessed

## Discussion

Tumor heterogeneity may have profound influence on cancer progression, as well as on our interpretation of prognostic and predictive biomarkers. In this study, we analyzed tumor heterogeneity based on high-resolution DNA copy number profiles of multiple liver deposits obtained from patients with metastatic CRC. Previous studies of metastatic CRC have focused on comparing primary tumors and individual metastatic deposits[16–19,25,30,31], or assessed prognostic impact of DNA copy number aberrations in primary tumors from patients with metastatic disease exposed to chemotherapy[14]. Here, we describe for the first time large variation among patients with respect to intra-patient inter-metastatic heterogeneity. Such heterogeneity is a natural outcome of the branched evolutionary pattern recently described in metastatic progression of CRC[15]. Reinforcing the clinical relevance of this biological process, inter-metastatic heterogeneity was found to be a stronger predictor of patient survival after liver resection than known clinicopathological parameters for metastatic CRC. There is evidence indicating intra-tumor heterogeneity in primary tumors to be associated with poor prognosis in different cancer forms[42,47–50]. However, to the best of our knowledge, this is the first study to explore the relationship between inter-metastatic genomic heterogeneity and survival in patients with colorectal liver metastases. The clinical management of metastatic CRC is complex [51]and large patient series are needed to address the clinically relevant subgroups. In this study, all patients have been treated with liver resection, but the number of patients available for group comparisons is limited. Still, the clinical relevance discovered here strongly suggests that inter-metastatic heterogeneity is a key feature to explore in future patient cohorts.

To summarize the level of inter-metastatic heterogeneity per patient, we calculated a heterogeneity score based on the Euclidean distance of copy numbers of individual genomic regions between pairs of samples. This approach retains the high resolution and genome-wide nature of the copy number data, and is similar to a method previously used to predict progression from a premalignant condition to adenocarcinoma of the esophagus [39]. Heterogeneity was also calculated as the average pair-wise proportion of the genome of the metastases with different copy numbers. There was a strong consistency between our results generated by the two different methods, underlining the robustness of the Euclidean distance-based method. The patient-wise measure was not associated with the number of metastases analyzed per patient, further supporting its suitability as a parameter of genetic heterogeneity at the DNA copy number level.

Low and high levels of genomic instability, defined as the proportion of the genome affected by DNA copy number changes below 25% and above 75% respectively, was recently shown to be associated with a reduced risk of mortality in a large pan-cancer analysis of primary tumors [42]. Here, we identify the same favorable prognostic association in metastatic CRC; designated as a low patient-wise level of genomic complexity (there were no patients with a level of genomic complexity higher than 74%). This is in accordance with the well-established link between the chromosomal instability phenotype and poor patient prognosis in CRC[8,52]. Interestingly, the level of intra-patient inter-metastatic heterogeneity at the DNA copy number level was independent of the patient-wise average genomic complexity across metastatic deposits. The finding that high genomic variation among metastatic deposits may occur also when the tumors do not have a large proportion of the genome affected by copy number aberrations suggests that variation is not a random event, but related to key events during evolution of the individual tumors. Noteworthy, while the prognostic significance of inter-metastatic heterogeneity was independent of genomic complexity, the two factors in concert resulted in identification both of a patient subgroup with a particularly good and a subgroup with a particularly poor survival. This strengthens the notion that genomic complexity and inter-metastatic heterogeneity represent independent and key biological features of metastatic CRC.

There were indications of increased intra-patient inter-metastatic heterogeneity in the total patient series after exposure to chemotherapy; however, this difference was largest among patients harboring metachronous metastases. Notably, this effect was corroborated in longitudinal analysis by comparison of deposits collected at first and second liver resections, albeit with small sample numbers. While previous findings have indicated a biological difference between synchronous and metachronous liver metastases[53], we currently lack an explanation for this discrepancy. Considering also the limited number of patients available for the analyses, validation studies are needed. Interestingly, the effect of chemotherapy exposure on metastatic heterogeneity in patients with metachronous metastases was not related to time between chemotherapy exposure and liver resection, as no difference in heterogeneity between deposits collected from patients treated with adjuvant chemotherapy (years prior to liver resection) and deposits from patients having pre-operative treatment (just prior to liver resection) was recorded. While analysis of the same metastatic deposits before and after chemotherapy exposure was not possible, a possible explanation for the effect of chemotherapy on heterogeneity is offered by successful eradication of large and genetically similar subclones, resulting in greater impact of smaller and genetically diverse subclones in the heterogeneity analyses, as well as potential expansion of these subclones. Among the genomic regions having a high level of copy number heterogeneity in metastases after exposure to chemotherapy was a region on chromosome arm 20p, encoding the gene *MACROD2*. This region has previously been shown to have increased genomic instability in cancer and to resemble common fragile sites[54,55]. The frequent occurrence of both gains and losses in this region, in particular after chemotherapy exposure, suggests this to be an unstable sequence rather than a target for cancer progression.

In conclusion, we describe for the first time a large variation among patients in the level of inter-metastatic heterogeneity at the DNA copy number level in CRC. Intra-patient inter-metastatic heterogeneity was found to be a biological feature independent of the number and genomic complexity of the metastases. Underlining the biological importance of heterogeneity in tumor evolution and reinforcing its clinical relevance in metastatic CRC, intra-patient inter-metastatic heterogeneity was found to be a stronger prognostic factor than any known clinicopathological parameter for these patients.

## Materials and Methods

### Ethics statement

The study was approved by the Regional Committee for Medical Research Ethics–West Norway (2010/631), and all investigation was conducted according to the Declaration of Helsinki. All patients provided written informed consent.

### Patient material

Between August 2006 and February 2012, a total of 134 liver metastatic deposits were collected from 45 patients undergoing partial liver resection for synchronous or metachronous metastatic CRC according to a prospective study protocol. This included eleven samples collected at second surgical intervention of liver metastasis in eight patients. Snap-frozen tissue from the primary tumor was available for six of the patients (Fig 1).

All patients were enrolled at the time of liver surgery. The number and size of the metastatic deposits were radiologically assessed by a CT scan prior to surgery, and as many deposits as possible collected for research purpose. Tissue was sampled immediately following tissue reception in the operating theatre, and individual samples were snap-frozen in liquid nitrogen.

Baseline patient characteristics and treatment are summarized in Table 1. Patients were selected for peri-operative chemotherapy based on standard criteria, such as previous treatment, extent of the metastatic disease, general health condition, and Carcinoembryonic Antigen levels. The chemotherapy treatment contained fluorouracil, folinic acid, and oxaliplatin administered every second week (FOLFOX or Nordic FLOX) [44,45]. One patient was also treated with irinotecan before liver resection.

Each patient had a CT scan performed within four weeks prior to start of chemotherapy and before liver resection. Patients receiving pre-operative chemotherapy were evaluated for treatment response by CT scans after three and six treatment courses, according to the Response Evaluation Criteria in Solid Tumors (RECIST 1.1) [46]. All patients who received previous adjuvant chemotherapy for the primary tumor (n = 8), pre-operative chemotherapy for previous liver metastases (n = 1), and/or chemotherapy prior to the liver resection (n = 20) were considered chemotherapy exposed. Among the chemonaïve patients, four had previously received radiotherapy; three with concomitant low-dose chemotherapy (capecitabine) as a radio-sensitizer. These patients were not considered exposed to systemic chemotherapy.

Genomic DNA was extracted from all tumor samples using the QIAamp DNA Mini Kit (Qiagen, Hilden, Germany), according to the manufacturer’s instructions. MSI-status was determined for the six primary CRCs and one metastatic deposit per patient by analyses of the BAT25 and BAT26 markers, as previously described (Table 1) [56]. The tumors were considered to have MSI if one of the two markers showed an aberrant profile.

### DNA copy number analysis

All 140 tumor samples were analyzed for DNA copy number variation using the Affymetrix Genome-Wide Human SNP Array 6.0 (Affymetrix Inc., Santa Clara, CA). This array contains more than 1.8 million single nucleotide polymorphisms (SNPs) and non-polymorphic copy number probes, with a median inter-marker distance of less than 700 bases genome-wide. 500 ng of genomic DNA from each sample was individually processed and hybridized onto the array according to the Affymetrix SNP 6.0 Cytogenetics Copy Number Assay. Raw probe intensity data from scanned images of the arrays were stored in cell intensity (CEL) files by the Affymetrix Gene Chip Command Console software (version 1.0). Quality control of the individual CEL files was performed using the Affymetrix Genotyping Console software (version 4.1.4.840). All samples had quality control metrics above the recommended threshold (Contrast QC > 0.4). The microarray data can be accessed from the NCBI’s Gene Expression Omnibus (GEO) with the accession number GSE63490.

### Preprocessing of DNA copy number data

The raw CEL data files were preprocessed according to the PennCNV protocol [57] adapted for Affymetrix genotyping arrays [58]. This includes allele-specific signal extraction, quantile normalization, median polishing, and signal intensity generation using the Affymetrix Power Tools software, followed by calculation of total signal intensity values (Log R Ratio, LRR) per marker position, and GC-adjustment to account for genomic waves in signal intensities. HapMap samples previously analyzed on the SNP Array 6.0 (n = 270 individuals from four populations) [59], and integrated in the PennCNV-Affy software package, were used as reference for normalization (hapmap.quant-norm.normalization-target.txt) and LRR calculation (hapmap.genocluster). The Affymetrix GenomeWideSNP_6.cdf annotation file was used as provided with the software package. The genomic position for each marker was given according to the NCBI 36 (hg18) human genome assembly, as specified in the PennCNV-Affy location file (affygw6.hg18.pfb). However, genomic regions further described have been converted to the GRCh37 assembly (hg19) using the web interface of the UCSC Batch Coordinate Conversion (liftOver) tool with default parameters (http://genome.ucsc.edu/cgi-bin/hgLiftOver).

To identify continuous genomic regions with equal copy numbers from the intensity values of adjacent independent markers, single-sample segmentation was performed with the PCF algorithm implemented in the Bioconductor package copynumber[60]. The penalty parameter gamma was set to 100 and the minimum number of probes per segment, k_min_, was set to 5, both of which are within the recommended range. Before segmentation, winsorization was performed to limit extreme values, using default settings in the copynumber package. The resulting PCF-values per sample and segment are further referred to as copy number estimates for the indicated genomic regions, and are relative to the HapMap samples.

Individual segmentation of the samples resulted in genomic segments of varying sizes and breakpoint positions between the samples. The median number of segments per samples was 96.5 (range 47 to 346), with a median sample-wise average segment size of 29.8M base pairs (range 8.3M to 61.2M; Fig L in S2 Text). To obtain common segments and allow for comparisons among the samples, the segments were divided into shorter segments defined by all breakpoint positions across all samples, further referred to as atomic segments. These atomic or longest common segments were identified separately for all sample group comparisons. For comparison with the atomic segmentation-approach, patient-wise joint-sample segmentation was also done using the multipcf-function in the copynumber library, to perform simultaneous segmentation of all samples per patient. The single-sample atomic segmentation and patient-wise joint-sample segmentation approaches revealed highly concordant results for both the average patient-wise genomic complexity and intra-patient inter-metastatic genetic heterogeneity among patients (Spearman’s rank correlation 0.99 and 0.83, P = 1×10^−49^ and 3×10^−11^, respectively; Fig L in S2 Text).

DNA copy number estimates of ±0.1 were used as thresholds for calling copy number aberrations (gains and losses). For comparison, different thresholds for identification of DNA copy number aberrations (ranging from 0.05 to 0.2) were tested and found to yield highly proportional results with respect to the genomic complexityper sample (Table C in S3 Text).

Probes targeting the allosomes, control probes (n = 3,643), duplicate probes (one of the two probes covering overlapping genomic loci; n = 187), and probes (n = 6,668) mapping to regions with recurrent high frequency aberrations in non-cancer samples from several organs, were excluded from further analyses. The latter set of probes was considered to result in data of poor technical quality and was identified as probes targeting genomic regions (n = 33 regions of totally 29.4M base pairs) with aberration frequencies higher than 10% in non-cancer samples from each of six publically available SNP Array 6.0 datasets (downloaded from the GEO and including 222 samples from six different origins; breast, GEO accession number GSE32258; head and neck, GSE33229; kidney, GSE19949; lung, GSE36363; prostate, GSE18333; blood samples from rectal cancer patients, GSE3282).

Annotations (HGNC gene symbols) for genes encoded in specific genomic regions were retrieved from the BioMart project, using the getBM() function and the hsapiens_gene_ensembl dataset in the R implementation (biomaRt)[61].

The cancer cell fraction was estimated for each sample based on genome-wide copy number profiles applying the algorithm ASCAT [40].

### Intra-patient inter-metastatic genetic heterogeneity

Patients were compared with regard to intra-patient heterogeneity in DNA copy number among their liver metastases. For initial exploration and visualization of potential heterogeneity,hierarchical clustering analysis was performed for all 123 metastatic deposits collected at first liver resection (based on all atomic segments with variance higher than 0.03 across all samples, including 19% of the segments) using the R package pvclust[62]. Clustering was done by complete linkage, based on Euclidean distance metrics and including assessment of the uncertainty of each cluster by calculation of approximately unbiased probability values from multiscale bootstrap resampling (n = 1,000).

To quantify the level of heterogeneity between any two samples, pair-wise genetic distances based on genome-wide copy number estimates were calculated as the Euclidean distance between paired copy number segments. This measure represents the square root of the sum of squared differences in DNA copy number estimates of all individual segments between the two samples, and is a multidimensional measure with the number of dimensions equal to the number of segments included. To analyze only the most informative segments, only segments with variance higher than 0.03 across all samples in each comparison were included. To obtain a single, patient-wise measure of heterogeneity, and to account for the varying numbers of samples analyzed per patient, intra-patient inter-metastatic heterogeneity was subsequently calculated in two ways. First, in analyses of metastatic deposits from the first liver resection only, the average pair-wise Euclidean distance of all possible combinations of metastases from each patient was calculated (illustrated in Fig 2C for a patient with three metastatic deposits). Second, in longitudinal analyses of metastatic deposits from the first and second liver resections, the average pair-wise Euclidean distance of all possible combinations of the sequential samples from each patient was calculated. To investigate the potential effect of filtering of segments prior to calculation of the heterogeneity score, intra-patient heterogeneity among metastases from the first liver resection was calculated also based on segments with variance higher than 0.05 across all samples, as well as based on all segments (no filtering). Compared with the heterogeneity score obtained using the 0.03 filtering criteria, the results from both alternative calculations were highly concordant (Pearson correlation 0.8 and 0.9, P = 2×10^−10^ and P = 3×10^−20^, respectively). Furthermore, to investigate the potential effect of the estimated ploidy per sample, intra-patient inter-metastatic heterogeneity was also calculated based on ploidy-adjusted, absolute DNA copy number estimates obtained from ASCAT (no filtering of segments). There was a good correlation between the ploidy-adjusted and original patient-wise heterogeneity scores (Pearson correlation 0.7, P = 3×10^−6^; Fig M in S2 Text). The intra-patient inter-metastatic heterogeneity score does not consider DNA copy number neutral events; however, copy number neutral loss of heterozygosity (cnLOH) was a far less frequent event than copy number aberrations. Based on absolute DNA copy number profiles obtained from ASCAT, the median proportion of the genome (per cent of base pairs) with DNA copy number aberrations was 35%among the 123 liver metastatic deposits from the first liver resection, compared with only 3% for cnLOH (calculated from segments with copy number zero for one allele and two for the other allele; P = 8×10^−27^ from paired t-test). In comparison with the original patient-wise heterogeneity score, there was good correlation with the heterogeneity score based on ASCAT estimates and including cnLOH (Pearson correlation 0.7, P = 2×10^−6^). Analyzing the heterogeneity score including cnLOH as a continuous variable in Cox’s regression, a continuously increasing level of heterogeneity among patients was associated with a continuously decreasing three-year survival rate (with borderline statistical significance; P = 0.05 and 0.1 for OS and PFS respectively; Fig M in S2 Text).

As an alternative method to the Euclidean distance-based measure, an additional measure of intra-patient inter-metastatic heterogeneity was calculated as the average pair-wise proportion of the genome of the metastases with different copy numbers. As with the Euclidean distance-based measure, the proportion of the genome that is different (summarized from segments with differences in DNA copy number estimates larger than 0.1, and reported as the per cent of base pairs)was calculated for all possible pair-wise comparisons of metastases from each patient, and a patient-wise measure was obtained as the average proportion from these pair-wise comparisons.

### Genomic complexity

The genomic complexity of each metastatic deposit was measured as the proportion (per cent of base pairs) of the genome with aberrant copy numbers. A patient-wise measure of genomic complexity was calculated as the average genomic complexity of all liver metastases analyzed per patient.

### *TP53* mutation screening

*TP53* mutation analysis was performed for the whole coding region of the gene in all liver metastatic deposits. *TP53* was amplified in a nested PCR and the PCR products were purified and sequenced on an automated ABI 3700 DNA sequencer (Applied Biosystems, Carlsbad, CA, U.S.A.; further detailed in Methods in S1 Text). All mutations identified were verified by exon-wise sequencing of genomic DNA.

### Statistical analyses

The patient-wise measures of inter-metastatic DNA copy number heterogeneity were compared among patients by independent or paired samples t-tests using the SPSS software version PASW 18.0 (IBM Corporation, Armonk, NY, U.S.A.). Two-sided P-values < 0.05 were considered significant. Individual segments with large DNA copy number variation were also identified by independent samples t-tests (unequal variances assumed), and P-values were adjusted for multiple comparisons using the p.adjust() function in R (reporting q-values from the Benjamini and Hochberg correction method).

Univariable and multivariable survival analyses were conducted with Cox’s proportional hazards regression, with calculation of P-values from Wald’s tests for predictive potential (SPSS). The proportional hazards assumption was tested by the cox.zph function in the survival package in R and found to be true (P-values from the chi-square test were higher than 0.3). In multivariable analyses, all variables found to have prognostic associations in univariable analyses were entered into the model in one single step. Kaplan-Meier survival curves were compared with the log-rank test. Three-year PFS (considering relapse or death from any cause as events, and censoring in the case of no event within three years) and OS (time to death from any cause) were used as endpoints. The time to event or censoring was calculated from the start of treatment for the liver metastases (liver resection or pre-operative chemotherapy).

## Supporting Information

S1 Text Supporting Results and Methods. (DOCX)

S2 Text Figures A-N. (DOCX)

S3 Text Tables A-E. (DOCX)
