# Supplementary material for: Intra-patient Inter-metastatic Genetic Heterogeneity in Colorectal Cancer as a Key Determinant of Survival after Curative Liver Resection
Source: PLoS Genet. 2016 Jul 29;12(7):e1006225. doi: 10.1371/journal.pgen.1006225 (PMC4966938; doi:10.1371/journal.pgen.1006225)
Supplement: S1 Text — (DOCX) [file pgen.1006225.s001.docx]

S1 Text

Supporting Results and Methods

**Intra-patient Inter-metastatic Genetic Heterogeneity in Colorectal Cancer as a Key Determinant of Survival after Curative Liver Resection**

Anita Sveen, Inger Marie Løes, Sharmini Alagaratnam, Gro Nilsen, Maren Høland, Ole Christian Lingjærde, Halfdan Sorbye, Kaja Christine Graue Berg, Arild Horn, Jon-Helge Angelsen, Stian Knappskog, Per Eystein Lønning, Ragnhild A. Lothe

# Results

## DNA copy number aberration frequencies in primary colorectal tumors and liver metastatic deposits

A limited number of primary tumors (n = 6) were available for comparison with the liver metastases. However, in an extended comparison of all the liver metastases (n = 134) with a publicly available dataset of primary CRCs (n = 62 CRCs analyzed on the SNP Array 6.0; downloaded from the NCBI’s Gene Expression Omnibus, accession number GSE36458), the two sample types showed similar aberration frequencies genome-wide. For the liver metastases, the frequencies were summarized patient-wise, and each patient was considered to have an aberration if found in at least one metastatic deposit. High frequencies of gain were found on chromosome arms 7p,7q, 8q, 13q, and 20q, and high frequencies of loss on 1p, 4p, 4q, 8p, 17p, and 18q (Fig A in S2 Text).

## Response to pre-surgical chemotherapy and DNA copy number aberrations in liver metastases

Among the patients receiving pre-operative chemotherapy before the first liver resection (n = 20), there were two response groups, stable disease and partial response (evaluated according to RECIST 1.1). There was no difference in the level of intra-patient inter-metastatic genetic heterogeneity between the two response groups (Fig G in S2 Text). Similarly, analyzing patients with metachronous and synchronous metastases separately, no difference in heterogeneity between the two response groups was observed (Fig G in S2 Text). However, there were several genomic regions with large differences in the frequency of DNA copy number aberrations between the two response groups (summarized from patients with aberrations in at least one metastatic deposit; Table D in S3 Text). For many of these regions, gains in tumors from patients with stable disease were associated with a concomitant loss in patients with partial response (Fig N in S2 Text and Table E in S3 Text). In particular, this was the case for regions on 9p. The same association was not seen in the reverse setting (high frequency of loss in patients with stable disease was associated with a low aberration frequency in patients with partial response).

## Genomic regions with high intra-patient variance in copy number after exposure to chemotherapy

To identify the genomic regions with the greatest difference in intra-patient heterogeneity between patients with metachronous metastases who were exposed (n = 13) and non-exposed (n = 9) to chemotherapy, the variance in DNA copy number estimates among all metastatic deposits from each patient was calculated for all atomic segments separately, and then compared between the two patient groups (independent samples t-tests). Among the ten most significantly different regions (all with highest variance in the chemotherapy exposed patients) were regions on chromosome arms 8q, 9q, 10q, 19p, and 20p (Table B in S3 Text). None of the regions had significantly different variance between the treatment groups after correcting for multiple comparisons.

# Methods

## *TP53* mutation screening by capillary sequencing

*TP53* mutation analysis was performed for the whole coding region of the gene on complementary DNA (cDNA) from all liver metastatic deposits. RNA was extracted from fresh frozen tissue using the RNeasy Mini Kit (Qiagen, Hilden, Germany), and cDNA was synthesized using qScript cDNA SuperMix (Quanta, BioSciences, Gaithersburg, MD, U.S.A.). Both procedures were performed according to the manufactures’ instructions. *TP53* was amplified in a nested PCR. The first round of amplification was performed using Dynazyme EXT DNA polymerase (FINNZYMES, Espoo, Finland) in a 50 μl reaction mix containing 1x PCR buffer, 1.5 mM MgCl_2_, 0.2 mM of each deoxynucleotide triphosphate, 5% DMSO, 0.2 μM of each primer (5’-*gacactttgcgttcgggc*-3’and 5’-*cttgttcagtggagccccg*-3’) and 0.5 µl cDNA template. The thermocycling conditions were an initial denaturation step of 5 min at 94⁰C followed by 40 cycles of 30 sec at 94⁰C, 30 sec at 51⁰C and 2 min at 72⁰C, before a final elongation step of 7 min at 72⁰C. The second round of amplification was performed using identical thermocycling conditions and reaction mix as the first amplification round, but using nested primers (5’-*gacacgcttccctggattgg*-3’ and 5’-*cgcacacctattgcaagcaaggg*-3’) and 1 µl of the first round product as template. Prior to sequencing, PCR products were purified using the ExoSAP-IT kit (GE Healthcare) according to the manufacturer’s instructions. Sequencing was done using BigDye version 1.1 cycle sequencing Kit (Applied Biosystems, Carlsbad, CA, U.S.A.) according to the manufacturer’s instructions, with specific internal sequencing primers (5’-*tggcccctcctcagcatctta*-3’ and 5’-*ggtacagtcagagccaacctc*-3’). Thermal conditions were 30 cycles of denaturation at 94°C for 15 seconds, annealing at 50°C for 5 seconds and elongation at 60°C for 4 minutes. Capillary electrophoresis, data collection, and sequence analysis were performed on an automated DNA sequencer (ABI 3700, Applied Biosystems). For some of the samples, the number of cycles in the nested PCR was adjusted, due to the presence of several uncharacterized products of distinct sizes precluding the sequences. All mutations identified were verified by exon-wise sequencing of genomic DNA.
