# Supplementary material for: Intra-patient Inter-metastatic Genetic Heterogeneity in Colorectal Cancer as a Key Determinant of Survival after Curative Liver Resection
Source: PLoS Genet. 2016 Jul 29;12(7):e1006225. doi: 10.1371/journal.pgen.1006225 (PMC4966938; doi:10.1371/journal.pgen.1006225)
Supplement: S2 Text — (DOCX) [file pgen.1006225.s002.docx]

S2 Text

Figures A-N

**Intra-patient Inter-metastatic Genetic Heterogeneity in Colorectal Cancer as a Key Determinant of Survival after Curative Liver Resection**

Anita Sveen, Inger Marie Løes, Sharmini Alagaratnam, Gro Nilsen, Maren Høland, Ole Christian Lingjærde, Halfdan Sorbye, Kaja Christine Graue Berg, Arild Horn, Jon-Helge Angelsen, Stian Knappskog, Per Eystein Lønning, Ragnhild A. Lothe


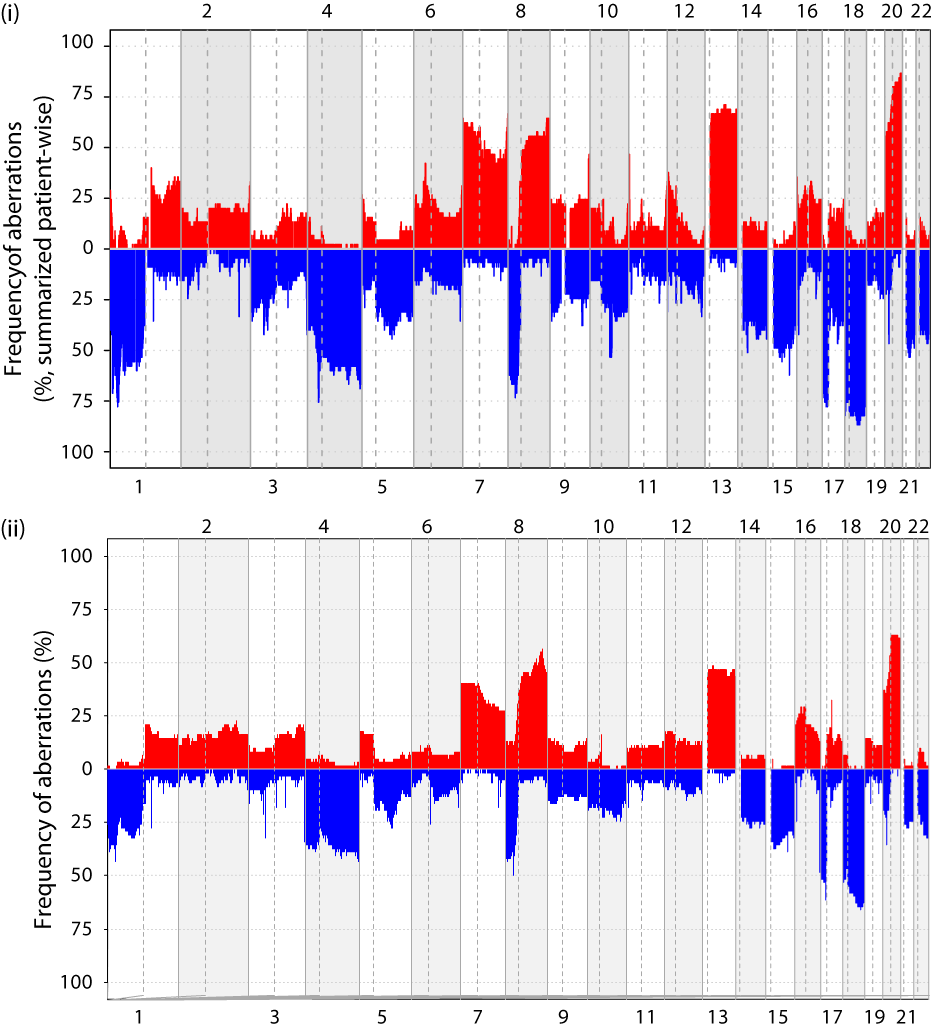


# Fig A. Frequency of copy number aberrations

Copy number profiles of (i) liver metastases analyzed in this study (n = 134 from 45 patients; frequencies were summarized patient-wise) and (ii) primary CRCs (n = 62; publicly available data, GEO accession number GSE36458) show similar frequencies (vertical axes) of gains (red) and losses (blue) along the genome (chromosome numbers are indicated on the horizontal axes; chromosomes and chromosome arms are separated by vertical lines). High-frequency aberrations that have previously been described in CRC are found in both datasets, including gains on chromosome arms 7p, 7q, 8q, 13q, and 20q, as well as losses on 1p, 4p, 4q, 8p, 17p, and 18q.


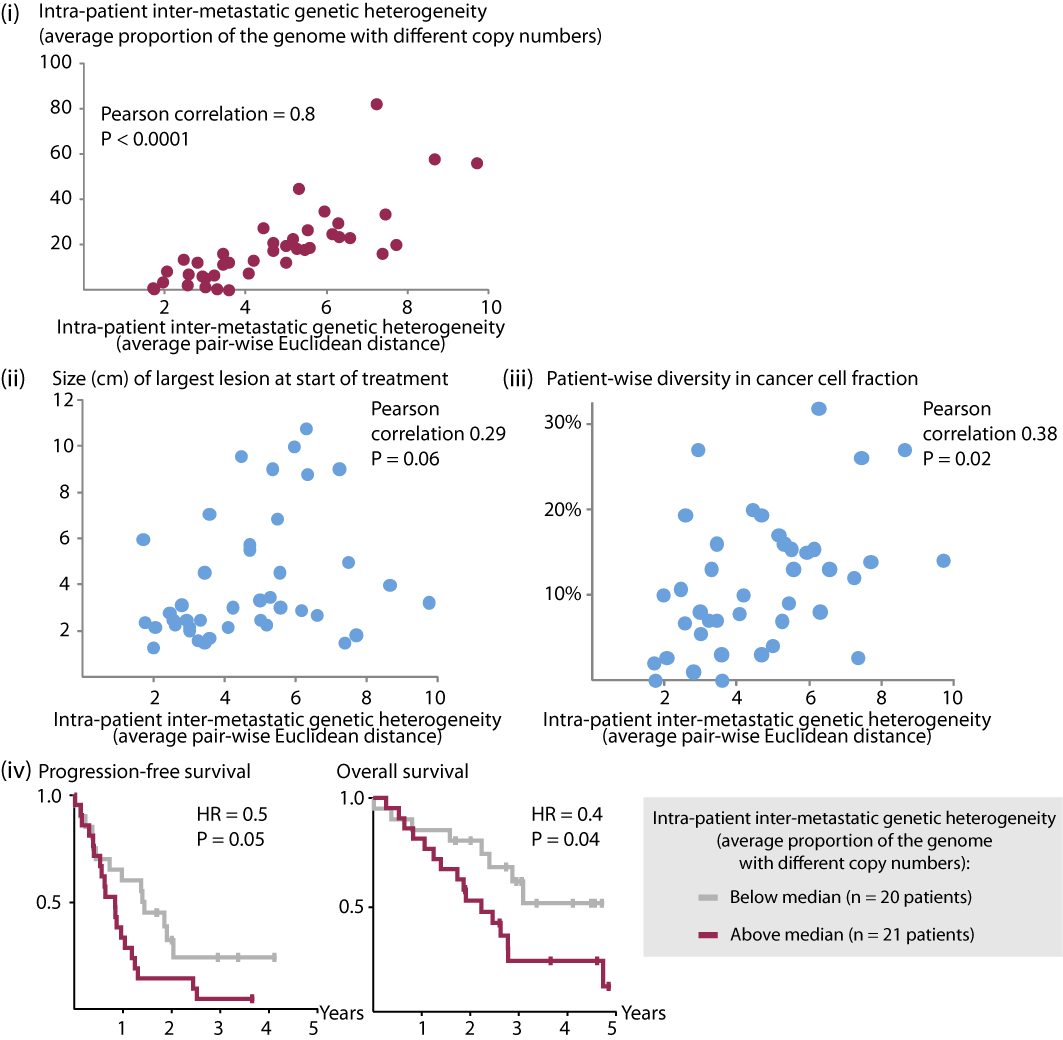


# Fig B. Alternative measure of intra-patient inter-metastatic genetic heterogeneity and the patient-wise diversity in cancer cell fraction

(i) As an additional measure of intra-patient inter-metastatic genetic heterogeneity, the average pair-wise proportion of the genome of the metastases with different copy numbers was calculated (summarized from segments with larger than 0.1 difference in copy number estimates between pairs of metastases, and reported as the per cent of base pairs). This measure corresponded well with intra-patient heterogeneity calculated as the average pair-wise Euclidean distance of the genome-wide copy numbers. (ii) There was little association between the size of the largest metastatic deposit per patient and intra-patient inter-metastatic genetic heterogeneity. (iii) The cancer cell fraction of the metastatic samples was estimated using ASCAT (ASCAT was unable to estimate the cancer cell fraction of six samples). The patient-wise diversity in the cancer cell fraction (calculated as the absolute difference in the cancer cell fraction of all possible pair-wise comparisons of metastatic samples from each patient) was only weakly associated with intra-patient inter-metastatic genetic heterogeneity. The patient-wise diversity in cancer cell fraction could be calculated for 39 of the 41 patients with multiple metastatic deposits. (iv) The alternative measure of intra-patient inter-metastatic genetic heterogeneity was associated with patient outcome (hazard ratios, HR, at three years were calculated by Cox’s regression and P-values from Wald’s tests of predictive potential).


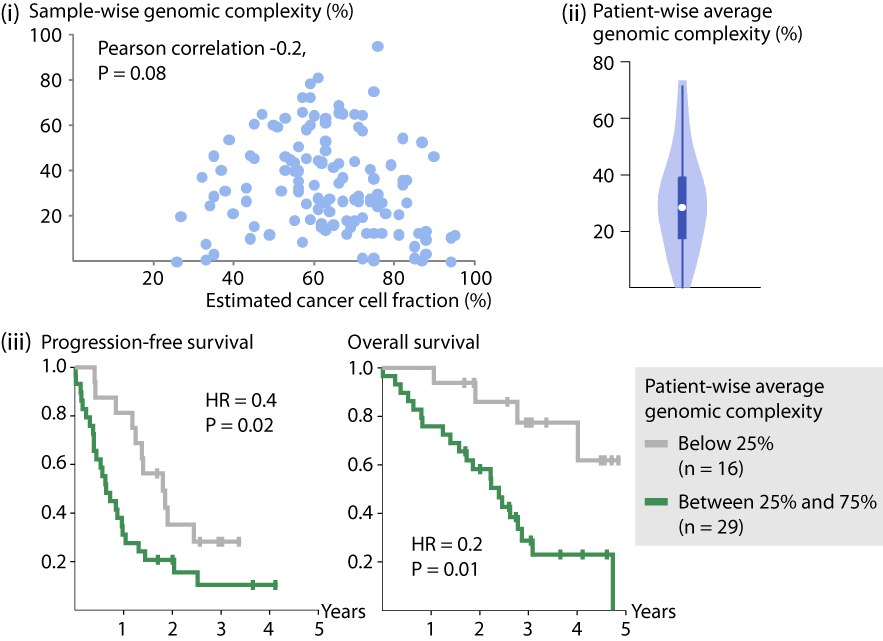


# Fig C. Genomic complexity is independent of the tumor cell purity of the samples and associated with patient survival

Genomic complexity was calculated for each metastatic deposit (n = 134) as the proportion of the genome (per cent of base pairs) with aberrant copy number. (i) The sample-wise genomic complexity was not associated with the sample-wise cancer cell fraction, as estimated by ASCAT. (ii) A patient-wise measure of genomic complexity was calculated as the average genomic complexity of all liver metastases collected from the first liver resection of each patient. This ranged from 0 to 74% (median 28%). (iii) Patients with a low level of genomic complexity (below 25%) had a significantly better three-year progression-free and overall survival rate than patients with a genomic complexity between 25% and 75%, measured from start of treatment for the metastases. Hazard ratios (HR) at three years were calculated by Cox’s regression and P-values from Wald’s tests of predictive potential.


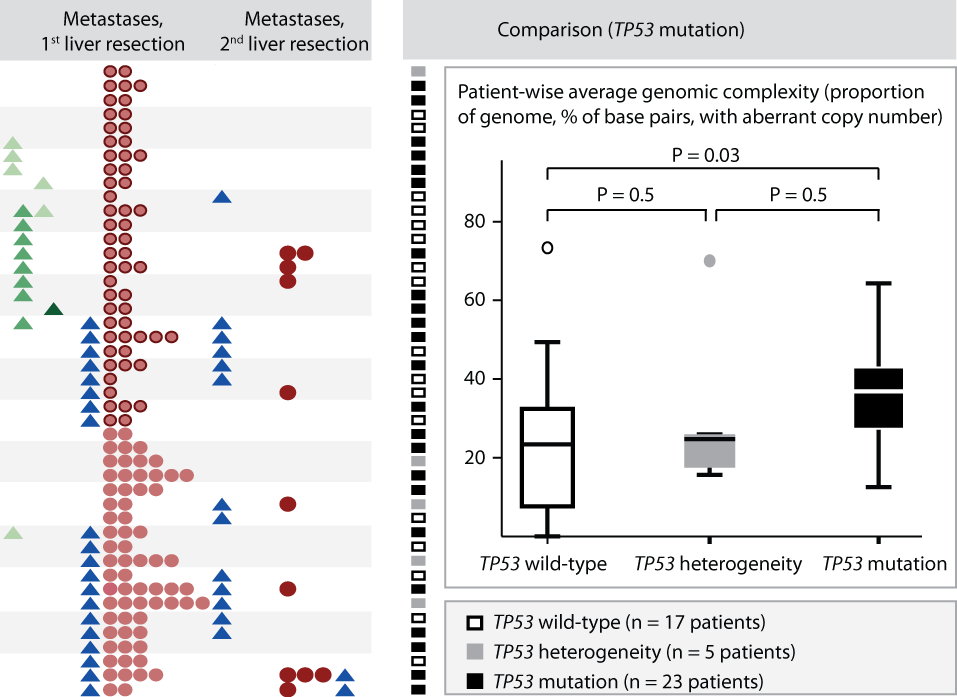


# Fig D. High genomic complexity in liver metastases with *TP53* mutations

The left panel illustrates the patients (vertically; n = 45) and samples (horizontally; n = 123 and 11 metastatic deposits collected at first and second liver resections respectively) analyzed (the symbols are the same as described in Fig 1). The right panel shows the result from comparison (independent samples t-tests) of the average genomic complexity between patients with either wild-type (n = 17; indicated by white boxes in the left part of the right panel), mutated (n = 23; indicated by black boxes), or heterogeneously mutated *TP53* (n = 5, four patients with both *TP53* mutated and wild-type metastases from the first liver resection and one patient with mutational heterogeneity between metastases collected at first and second liver resection; indicated by grey boxes).There was a significantly higher complexity in metastases with *TP53* mutations compared with wild type. Patients with heterogeneously mutated metastases had an intermediate level of genomic complexity; however, there was no significant difference compared to the patients with either wild type *TP53* or *TP53* mutations. Genomic complexity was measured patient-wise as the average proportion of the genome (per cent of base pairs) of metastatic deposits with aberrant copy number.


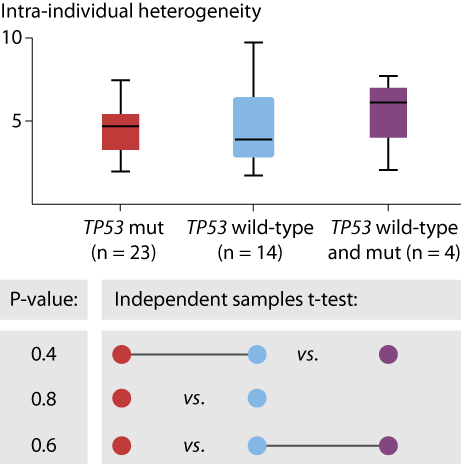


# Fig E. *TP53* mutations and intra-patient inter-metastatic heterogeneity

There was no difference in intra-patient inter-metastatic heterogeneity at the DNA copy number level between patients that were homogenous (all metastases either wild-type or mutated) and heterogenous (both wild-type and mutated metastases) for *TP53*. Also, no difference in heterogeneity between patients with *TP53* mutated and wild-type metastases was recorded. For this comparison with intra-patient heterogeneity, only metastatic deposits from first liver resection were analyzed, and here, four patients displayed mutational heterogeneity.


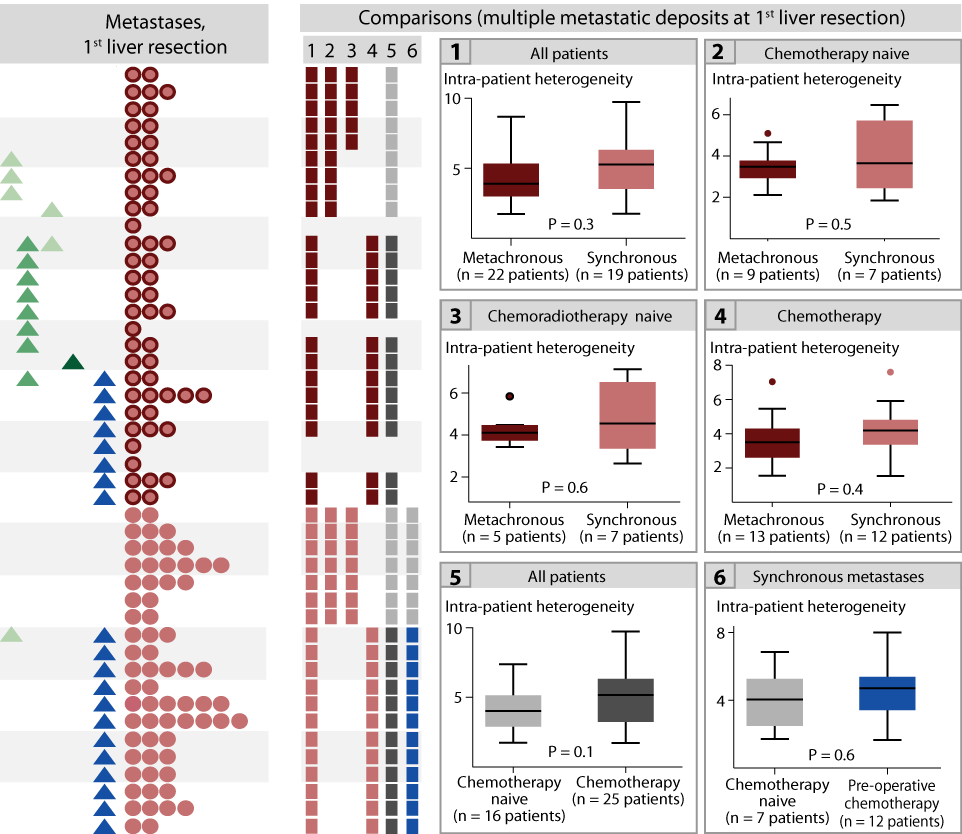


# Fig F. Intra-patient inter-metastatic heterogeneity, synchronous or metachronous presentation of metastases and previous chemotherapy exposure

Forty-one of the totally 45 patients (vertically in the left panel) had two or more liver deposits (horizontally in the left panel) at first liver resection (the symbols are the same as described in Fig 1). The right panel (right part, boxes 1-6) shows the results from comparisons (indicated by colored boxes in the left part of the right panel; independent samples t-test) of intra-patient inter-metastatic genetic heterogeneity between different patient groups. There was little difference in heterogeneity between patients with metachronous and synchronous liver metastases, either when analyzing all 41 patients (comparison 1), chemotherapy naïve patients only (including and excluding patients treated with radiotherapy or chemoradiotherapy; comparisons 2 and 3), or patients treated with chemotherapy only (comparison 4). Among all 41 patients, there were indications of higher heterogeneity in patients treated with chemotherapy than in chemonaïve patients (statistically non-significant; comparison 5). For patients with synchronous metastases only, there was no increase in heterogeneity after exposure to chemotherapy (comparison 6).


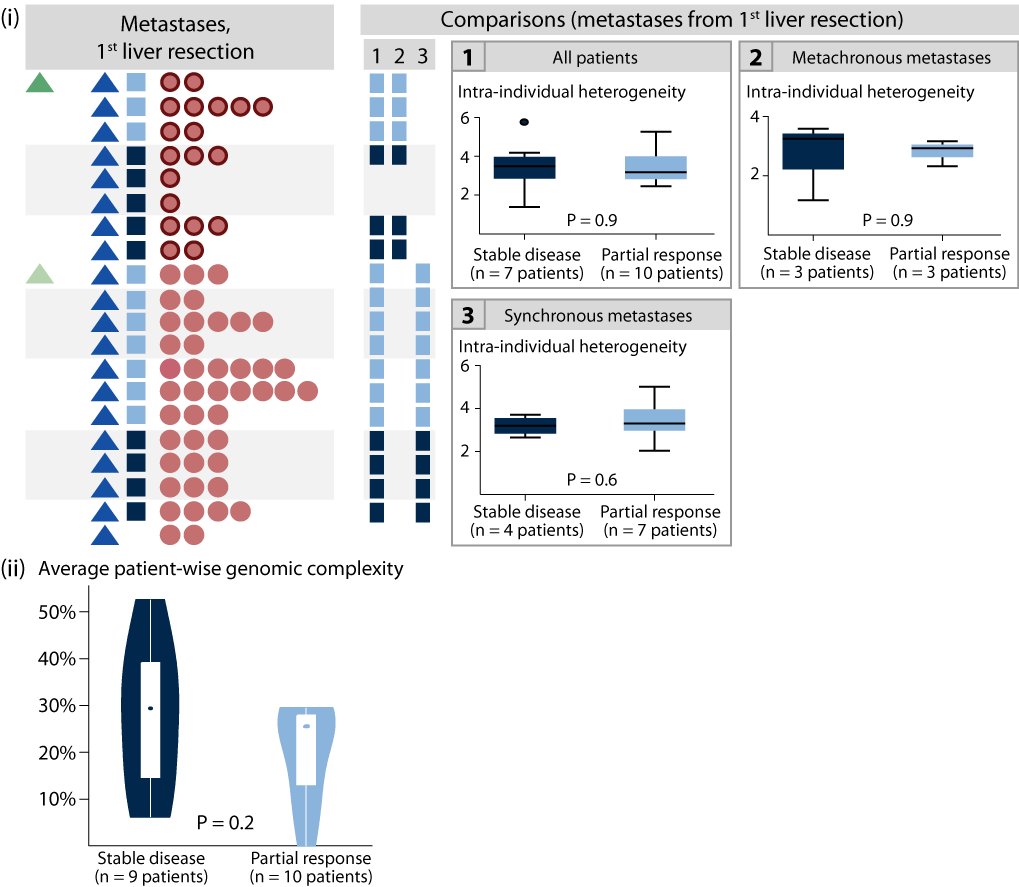


# Fig G. Comparisons of intra-patient inter-metastatic heterogeneity and genomic complexity between patient groups with different responses to chemotherapy

(i) The left panel illustrates the patients receiving pre-operative chemotherapy before the first liver resection (n = 20; the symbols are the same as described in Fig 1). The right panel (right part, boxes 1-3) shows the results from comparisons (indicated by colored boxes in the left part of the right panel; independent samples t-test) of intra-patient inter-metastatic genetic heterogeneity between patients that had either stable disease or partial response after chemotherapy (evaluated according to the RECIST 1.1 criteria). There was no difference in heterogeneity between the response groups, neither for all the patients combined (comparison 1), nor for patients with either metachronous (comparison 2) or synchronous metastases (comparison 3). (ii) Also with respect to the average patient-wise genomic complexity, there was no significant difference between the two response groups.


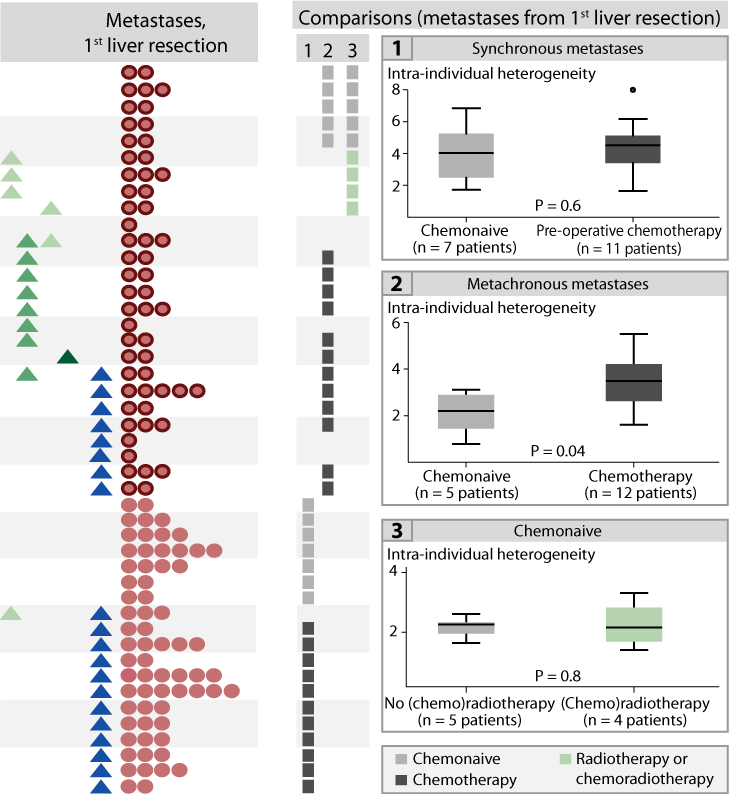


# Fig H. Comparisons of intra-patient inter-metastatic heterogeneity excluding patients treated with chemoradiotherapy

Forty-one of the totally 45 patients (vertically in the left panel) had two or more liver deposits (horizontally in the left panel) at first liver resection (the symbols are the same as described in Fig 1). The right panel (right part, boxes 1-3) shows the results from comparisons (indicated in the left part of the right panel; independent samples t-test) of intra-patient inter-metastatic genetic heterogeneity between different patient groups. Excluding patients treated with radiotherapy or chemoradiotherapy (total n = 6), there was no difference in heterogeneity between patients with synchronous metastases exposed or non-exposed to chemotherapy (comparison 1). However, among patients with metachronous metastases, heterogeneity was higher among patients exposed to chemotherapy (comparison 2). There was no difference in heterogeneity among chemonaïve patients and patients previously exposed only to radiotherapy or concomitant chemoradiotherapy (comparison 3).


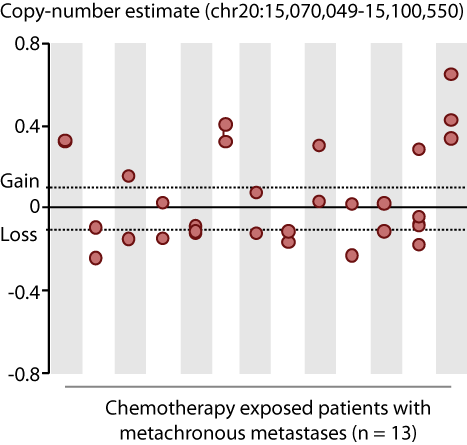


# Fig I. Genomic region with high intra-patient variance in copy number after exposure to chemotherapy

A region on chromosome 20p, partly encoding the gene *MACROD2*, showed high intra-patient variation in copy number (vertical axis) among metastatic deposits (metachronous, red dots) from patients (n = 13, horizontally) that had been treated with chemotherapy (patients treated with radiotherapy or chemoradiotherapy only were not included). The region was frequently both gained and lost (copy number estimates exceeding 0.1in absolute value).


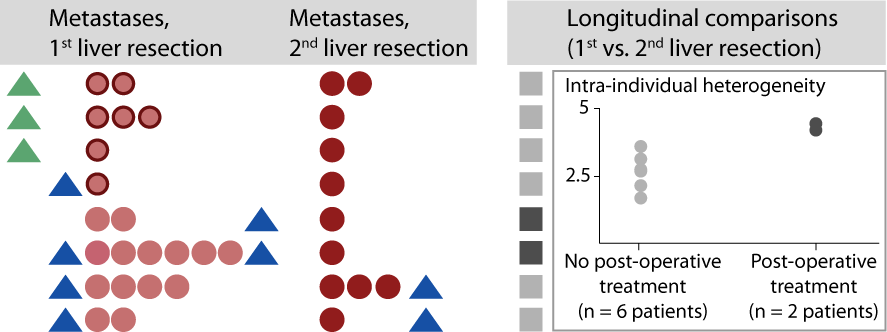


# Fig J. Longitudinal intra-patient heterogeneity between metastatic deposits from first and second liver resections

The left panel illustrates the patients (vertically; n = 8) and samples (horizontally; n = 21 and 11 metastatic deposits from the first and second liver resection respectively) analyzed (the symbols are the same as described in Fig 1). The right panel shows the comparison of longitudinal intra-patient inter-metastatic heterogeneity between metastatic deposits collected at first and second liver resection. Patients that received chemotherapy after the first resection (indicated by dark grey boxes in the left part of the right panel) had increased heterogeneity compared with patients that did not receive chemotherapy in this setting (indicated by light grey boxes).

**
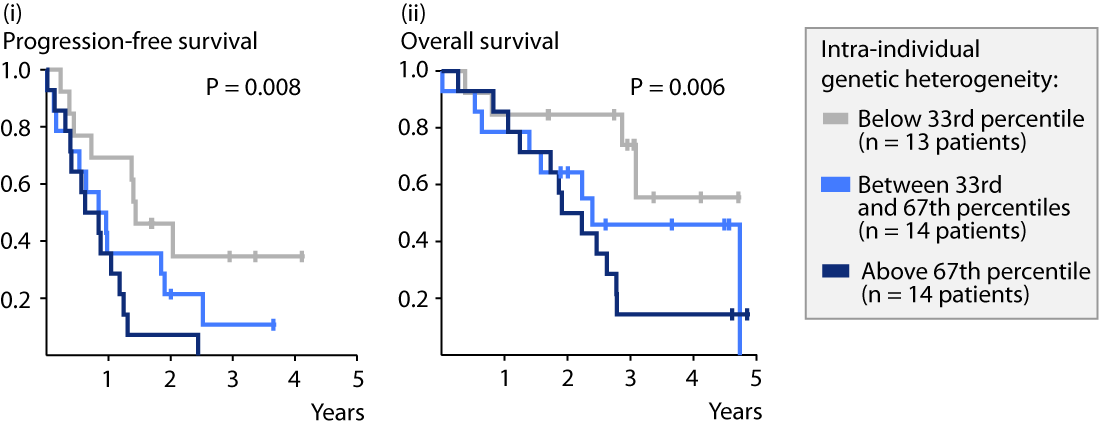
**

# Fig K. Intra-patient heterogeneity and patient survival

Separating the patients in three groups based on the 33^rd^ and 67^th^ percentiles of intra-patient inter-metastatic genetic heterogeneity, there was a linear trend for an increasingly favorable three-year (i) progression-free and (ii) overall survival rate with a decreasing level of heterogeneity (log-rank test of linear trend). Median progression-free survival was 17, 10 and 7 months for patients with a level of heterogeneity below the 33^rd^ percentile, between the 33^rd^ and 67^th^ percentiles, and above the 67^th^ percentile respectively. The corresponding three-year overall survival rates were 74%, 46% and 14%.


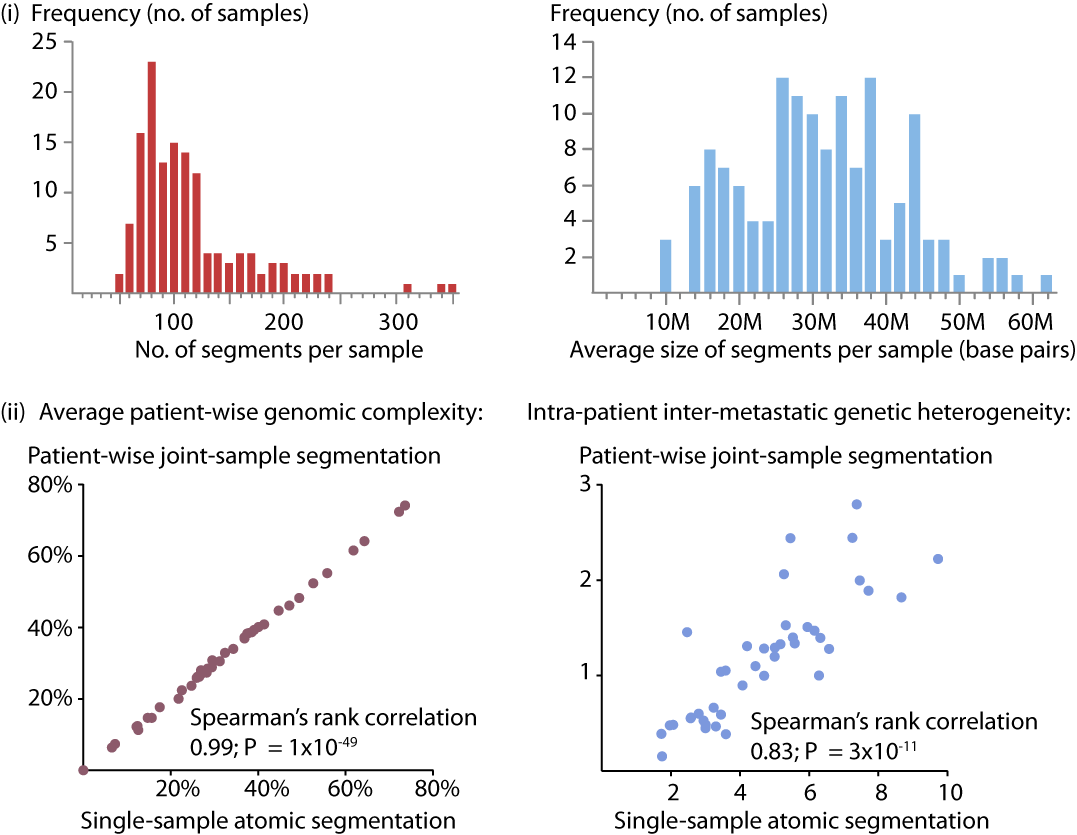


# Fig L. Individual sample and patient-wise joint-sample segmentation

(i) Single-sample segmentation of genome-wide copy number data for all the 140 samples resulted in a median of 96.5 (range 47 to 346) segments per sample and a median sample-wise average segment size of 29.8M base pairs (range 8.3M to 61.2M). (ii) Calculations of both the average patient-wise genomic complexity and intra-patient inter-metastatic genetic heterogeneity among patients based on single-sample segmentation *versus* patient-wise joint-segmentation revealed highly concordant results.


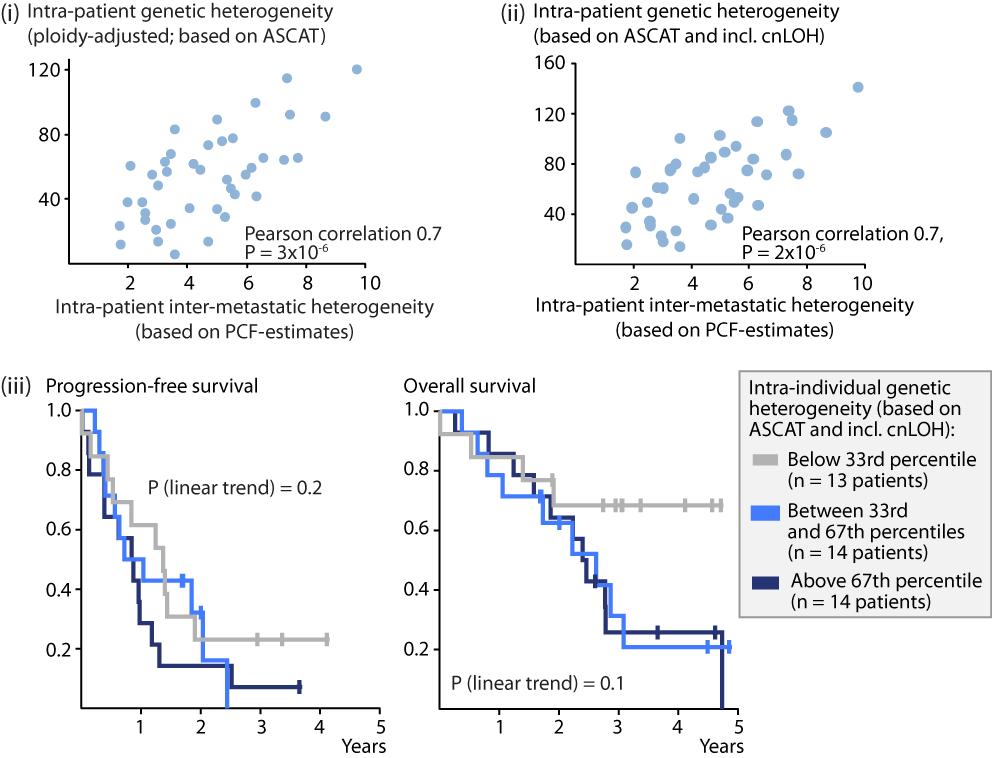


# Fig M. Intra-patient inter-metastatic genetic heterogeneity based on absolute copy number estimates from ASCAT

There was a good correspondence between the original intra-patient inter-metastatic heterogeneity score (calculated from PCF-estimates; horizontal axis) and the heterogeneity score based on absolute copy number estimates from ASCAT (vertical axis) including (i) ploidy-adjustment and (ii) cnLOH. (iii) A low patient-wise heterogeneity score (including cnLOH) was associated with a favorable patient outcome, although not statistically significant by log-rank tests of linear trend for the heterogeneity score divided into three patient groups as indicated. cnLOH, copy neutral loss of heterozygosity.


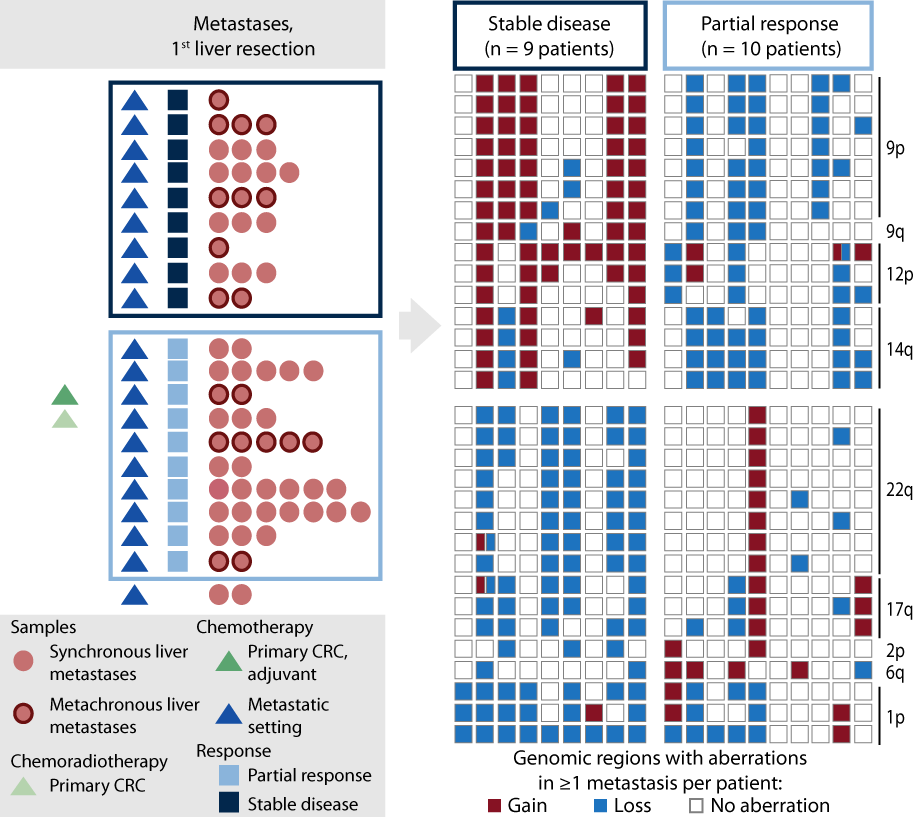


# Fig N. Copy number aberrations in patients with different response to pre-operative chemotherapy

Patients with different response to pre-operative chemotherapy (left panel) have several genomic regions with different frequencies of copy number aberrations in their metastases (right panel). Gain in patients with stable disease (at least one metastasis per patient) is associated with concomitant loss in patients with partial response (top right panel), but not *vice* *versa* (bottom right panel). In this plot, the order of the patients is the same vertically in the left panel (each row represents one patient) and horizontally in the right panel (each column represents one patient). In the right panel, each row represents a genomic region, with chromosome arms indicated to the right (and detailed further in Table D in S3 Text).
