# Supplementary material for: Intra-patient Inter-metastatic Genetic Heterogeneity in Colorectal Cancer as a Key Determinant of Survival after Curative Liver Resection
Source: PLoS Genet. 2016 Jul 29;12(7):e1006225. doi: 10.1371/journal.pgen.1006225 (PMC4966938; doi:10.1371/journal.pgen.1006225)
Supplement: S3 Text — (DOCX) [file pgen.1006225.s003.docx]

S3 Text

Tables A-E

**Intra-patient Inter-metastatic Genetic Heterogeneity in Colorectal Cancer as a Key Determinant of Survival after Curative Liver Resection**

Anita Sveen, Inger Marie Løes, Sharmini Alagaratnam, Gro Nilsen, Maren Høland, Ole Christian Lingjærde, Halfdan Sorbye, Kaja Christine Graue Berg, Arild Horn, Jon-Helge Angelsen, Stian Knappskog, Per Eystein Lønning, Ragnhild A. Lothe

# Table A. Genomic regions with increased frequency of copy number aberrations in metastases with mutated and wild type *TP53*

| **Chromosome** | **Start position** | **End position** | **Frequency of metastases with aberrations (%)** | |
| --- | --- | --- | --- | --- |
|  |  |  | **Metastases with *TP53* mutations (n = 89)** | **Metastases with wild-type *TP53* (n = 44)** |
| **Regions with gain in metastases with *TP53* mutations^a^:** | | | |  |
| 13 | 49,679,627 | 49,816,054 | 65 | 25 |
| 20 | 55,183,547 | 55,183,780 | 84 | 43 |
| 20 | 55,186,214 | 55,186,214 | 84 | 43 |
| 20 | 55,190,109 | 55,256,002 | 84 | 43 |
| 20 | 55,256,235 | 55,362,831 | 84 | 43 |
| 20 | 55,363,442 | 55,402,835 | 85 | 43 |
| **Regions with loss in metastases with *TP53* mutations^a^:** | | | |  |
| 1 | 10,657,912 | 12,119,569 | 61 | 20 |
| 1 | 12,125,536 | 16,368,403 | 62 | 20 |
| 1 | 33,614,162 | 33,880,813 | 61 | 18 |
| 1 | 57,793,355 | 58,834,609 | 61 | 18 |
| 1 | 58,834,962 | 58,834,962 | 61 | 20 |
| 4 | 91,418,248 | 91,472,419 | 63 | 23 |
| 4 | 91,473,208 | 91,473,208 | 63 | 23 |
| 4 | 91,475,787 | 91,504,762 | 63 | 23 |
| 4 | 91,577,260 | 91,630,126 | 63 | 23 |
| 4 | 91,746,551 | 91,747,456 | 63 | 23 |
| 4 | 91,747,664 | 91,789,526 | 64 | 23 |
| 4 | 91,792,568 | 91,919,051 | 64 | 23 |
| 4 | 91,919,084 | 92,005,908 | 64 | 23 |
| 4 | 92,011,445 | 92,066,814 | 64 | 23 |
| 4 | 92,426,319 | 92,477,472 | 64 | 23 |
| 4 | 92,477,566 | 92,488,837 | 64 | 23 |
| 4 | 92,491,825 | 93,620,783 | 64 | 23 |
| 4 | 93,621,793 | 94,127,660 | 64 | 23 |
| 4 | 94,129,968 | 95,049,123 | 64 | 23 |
| 4 | 108,188,671 | 108,758,579 | 63 | 23 |
| 4 | 108,761,386 | 108,761,386 | 63 | 23 |
| 4 | 108,762,717 | 108,915,125 | 63 | 23 |
| 4 | 108,918,564 | 108,918,564 | 63 | 23 |
| 4 | 108,918,849 | 109,611,560 | 63 | 23 |
| 4 | 110,975,433 | 113,118,942 | 64 | 23 |
| 4 | 114,159,052 | 114,217,620 | 64 | 23 |
| 4 | 114,217,997 | 114,217,997 | 64 | 23 |
| 4 | 114,218,202 | 114,786,698 | 64 | 23 |
| 4 | 114,792,093 | 114,797,011 | 64 | 23 |
| 4 | 114,797,242 | 115,825,926 | 64 | 23 |
| 4 | 115,826,311 | 115,832,552 | 63 | 23 |
| 4 | 135,047,355 | 135,537,968 | 64 | 23 |
| 4 | 135,543,476 | 135,543,476 | 63 | 23 |
| 4 | 137,864,196 | 138,179,445 | 64 | 23 |
| 4 | 138,182,433 | 138,190,884 | 67 | 25 |
| 4 | 138,191,970 | 138,191,970 | 66 | 23 |
| 4 | 138,192,024 | 138,254,638 | 64 | 23 |
| 4 | 138,254,846 | 138,558,565 | 63 | 23 |
| 4 | 143,853,943 | 144,242,990 | 65 | 25 |
| 4 | 144,679,032 | 148,492,727 | 64 | 23 |
| 4 | 148,500,176 | 148,500,176 | 63 | 23 |
| 4 | 178,973,203 | 179,204,774 | 65 | 25 |
| 4 | 179,205,396 | 179,775,385 | 65 | 23 |
| 4 | 180,696,728 | 181,103,729 | 65 | 23 |
| 18 | 3,791,294 | 3,791,294 | 78 | 36 |
| 18 | 3,793,080 | 3,808,500 | 78 | 36 |
| 18 | 3,812,319 | 3,839,680 | 78 | 34 |
| 18 | 3,840,072 | 3,930,317 | 78 | 34 |
| 18 | 3,933,975 | 4,620,374 | 78 | 34 |
| 18 | 4,628,275 | 5,966,350 | 78 | 36 |
| 18 | 5,967,110 | 6,748,937 | 78 | 32 |
| 18 | 6,755,963 | 6,762,861 | 78 | 34 |
| 18 | 6,763,039 | 7,037,428 | 78 | 36 |
| 18 | 7,037,639 | 9,096,036 | 78 | 36 |
| 18 | 9,097,460 | 9,464,919 | 78 | 36 |
| 18 | 9,465,903 | 10,493,908 | 78 | 36 |
| 18 | 10,494,381 | 11,277,057 | 78 | 36 |
| 18 | 11,277,464 | 13,140,123 | 78 | 36 |
| 18 | 13,142,971 | 14,090,993 | 78 | 36 |
| 18 | 14,091,511 | 14,113,928 | 78 | 34 |
| 18 | 14,115,121 | 14,115,121 | 78 | 34 |
| 18 | 14,123,194 | 14,144,453 | 78 | 34 |
| 18 | 14,144,777 | 15,402,408 | 76 | 34 |

^a^>40% difference in frequency of metastases with aberrations (TP53 mutation vs. wild-type)

# Table B. Genomic regions with increased copy number variance in response to chemotherapy

| **Chromosome** | **Start position** | **End position** | **P-value^a^** | **q-value^b^** | **No. of annotated genes** | **HGNC symbols** |
| --- | --- | --- | --- | --- | --- | --- |
| **Treated (n = 13; excluding patients treated with chemoradiotherapy only) *vs*. chemonaive (n = 9) patients with metachronous metastases^c^:** | | | | | | |
| 8 | 78,257,162 | 78,257,454 | 0.01 | 0.5 | - | *-* |
| 9^d^ | 112,780,395 | 112,817,268 | ≤0.01 | 0.5 | 2 | *AKAP2, PALM2-AKAP2* |
| 10 | 71,198,994 | 71,294,224 | 0.01 | 0.5 | 2 | *TMEM256P1, TSPAN15* |
| 19^d^ | 90,910 | 5,968,688 | 0.004 | 0.5 | 216 | *ABCA7, ABHD17A, ADAMTSL5, ADAT3, AES, AMH, ANKRD24, AP3D1, APBA3, APC2, ARID3A, ARRDC5, ATCAY, ATP5D, ATP8B3, AZU1, BSG, BTBD2, C19orf10, C19orf24, C19orf25, C19orf26, C19orf35, C19orf70, C19orf71, C19orf77, C2CD4C, CACTIN, CACTIN-AS1, CAPS, CATSPERD, CCDC94, CDC34, CELF5, CFD, CHAF1A, CICP19, CIRBP, CIRBP-AS1, CNN2, CREB3L3, CSNK1G2, CSNK1G2-AS1, DAPK3, DAZAP1, DIRAS1, DOHH, DOT1L, DPP9, DUS3L, EBI3, EEF2, EFNA2, ELANE, FEM1A, FGF22, FSD1, FSTL3, FTLP5, FUT3, FUT5, FUT6, FZR1, GADD45B, GAMT, GIPC3, GNA11, GNA15, GNG7, GPX4, GRIN3B, GZMM, HCN2, HMG20B, HMGB2P1, HMHA1, HSD11B1L, IZUMO4, JSRP1, KDM4B, KISS1R, KLF16, LINC01002, LINGO3, LMNB2, LONP1, LRG1, LSM7, MADCAM1, MAP2K2, MATK, MBD3, MED16, MEX3D, MFSD12, MIDN, MIER2, MIR1227, MIR1909, MIR3187, MIR4321, MIR4745, MIR4746, MIR4747, MIR637, MIR7-3, MIR7-3HG, MISP, MKNK2, MOB3A, MPND, MRPL54, MUM1, NCLN, NDUFA11, NDUFS7, NFIC, NMRK2, NRTN, OAZ1, ODF3L2, ONECUT3, OR4F17, OR4F8P, OR4G1P, OR4G3P, PALM, PCSK4, PIAS4, PIP5K1C, PLEKHJ1, PLIN3, PLIN4, PLIN5, PLK5, POLR2E, POLRMT, PPAP2C, PRR22, PRSS57, PRTN3, PTBP1, PTPRS, R3HDM4, RANBP3, RAX2, REEP6, REXO1, RN7SL121P, RN7SL202P, RN7SL226P, RN7SL477P, RN7SL528P, RN7SL626P, RN7SL84P, RN7SL866P, RNA5SP462, RNF126, RNU6-1076P, RNU6-1223P, RNU6-2, RNU6-9, RNU6-993P, RPL32P34, RPL36, RPS15, RPS15P9, RPS2P52, S1PR4, SAFB, SAFB2, SBNO2, SCAMP4, SEMA6B, SF3A2, SGTA, SH3GL1, SHC2, SHD, SIRT6, SLC39A3, SNORD37, SNRPEP4, SPPL2B, STAP2, STK11, TBXA2R, TCEB1P28, TCF3, THEG, THOP1, TICAM1, TIMM13, TINCR, TJP3, TLE2, TLE6, TMEM259, TMIGD2, TMPRSS9, TNFAIP8L1, TPGS1, UBXN6, UHRF1, UQCR11, VMAC, VN2R11P, WDR18, ZBTB7A, ZFR2, ZNF554, ZNF555, ZNF556, ZNF57, ZNF77, ZNRF4* |
| 20 | 15,070,049 | 15,100,550 | 0.007 | 0.5 | 1 | *MACROD2* |

^a^Independent samples t-test comparing intra-patient variance in metastases; ^b^P-value adjusted for multiple comparisons by the Benjamini-Hochberg method; ^c^Highest variance in patient group receiving treatment (adjuvant treatment for primary colorectal cancer or pre-operative treatment for the liver metastases); ^d^Genomic regions containing more than one atomic segment

# Table C. Comparison of genomic complexity among all 140 samples based on different thresholds for identification of DNA copy number aberrations

| Threshold PCF-values for identification of DNA copy number aberrations | Pearson correlation^a^ | P-value^a^ |
| --- | --- | --- |
| ±0.05 and ±0.075 | 0.94 | 9×10^-66^ |
| ±0.05 and ±0.1 | 0.85 | 1×10^-39^ |
| ±0.075 and ±0.1 | 0.94 | 9×10^-67^ |
| ±0.1 and ±0.125 | 0.93 | 2×10^-60^ |
| ±0.1 and ±0.15 | 0.84 | 1×10^-38^ |
| ±0.125 and ±0.15 | 0.95 | 1×10^-72^ |
| ±0.15 and ±0.175 | 0.94 | 1×10^-67^ |
| ±0.175 and ±0.2 | 0.94 | 2×10^-66^ |

^a^Calculated by comparing the genomic complexity (proportion of the genome with aberrant copy numbers) across all samples based on the two indicated threshold values

# Table D. Genomic regions with different frequencies of copy number aberrations in patients with different responses to pre-surgical chemotherapy

| **Chromosome** | **Start position** | **End position** | **No. of patients with aberration in at least one metastatic deposit** | |
| --- | --- | --- | --- | --- |
|  |  |  | **Patients with stable disease (n = 9)** | **Patients with partial response (n = 10)** |
| **Regions with gain in patients with stable disease:** | | |  |  |
| 1 | 154,436,195 | 155,984,802 | 5 | 1 |
| 1 | 155,986,799 | 156,228,060 | 5 | 1 |
| 9 | 46,587 | 4,163,335 | 5 | 0 |
| 9 | 4,163,463 | 6,012,102 | 5 | 0 |
| 9 | 6,012,734 | 6,168,335 | 5 | 0 |
| 9 | 6,169,135 | 6,268,922 | 5 | 0 |
| 9 | 6,270,908 | 6,899,149 | 5 | 0 |
| 9 | 6,900,898 | 6,965,101 | 5 | 0 |
| 9 | 6,965,750 | 6,969,585 | 5 | 0 |
| 9 | 6,969,634 | 7,305,031 | 5 | 0 |
| 9 | 7,306,383 | 8,258,659 | 5 | 0 |
| 9 | 8,258,921 | 14,785,224 | 5 | 0 |
| 9 | 14,785,294 | 14,849,045 | 5 | 0 |
| 9 | 14,850,060 | 15,032,733 | 5 | 0 |
| 9 | 15,033,952 | 15,739,131 | 5 | 0 |
| 9 | 15,742,241 | 19,060,937 | 5 | 0 |
| 9 | 19,064,604 | 19,315,386 | 5 | 0 |
| 9 | 19,315,923 | 20,860,495 | 5 | 0 |
| 9 | 20,868,287 | 20,917,493 | 5 | 0 |
| 9 | 20,920,713 | 26,241,963 | 5 | 0 |
| 9 | 26,242,038 | 26,248,563 | 5 | 0 |
| 9 | 26,256,181 | 26,263,018 | 5 | 0 |
| 9 | 26,265,534 | 26,434,494 | 5 | 0 |
| 9 | 26,435,047 | 30,329,758 | 5 | 0 |
| 9 | 30,330,466 | 31,929,553 | 5 | 0 |
| 9 | 31,930,138 | 31,972,254 | 5 | 0 |
| 9 | 31,972,671 | 32,175,970 | 5 | 0 |
| 9 | 32,179,169 | 36,105,264 | 5 | 0 |
| 9 | 36,110,749 | 39,110,886 | 5 | 0 |
| 9 | 71,006,575 | 76,151,597 | 4 | 0 |
| 9 | 76,151,707 | 76,273,471 | 4 | 0 |
| 9 | 76,273,853 | 80,029,750 | 4 | 0 |
| 9 | 80,029,803 | 80,029,803 | 4 | 0 |
| 9 | 80,032,300 | 86,638,977 | 4 | 0 |
| 9 | 86,639,109 | 100,380,227 | 5 | 0 |
| 9 | 100,381,034 | 101,191,784 | 5 | 1 |
| 9 | 101,194,716 | 101,525,021 | 5 | 0 |
| 9 | 101,527,665 | 104,943,675 | 5 | 1 |
| 9 | 135,767,171 | 135,767,171 | 6 | 2 |
| 9 | 135,767,185 | 135,767,312 | 6 | 2 |
| 9 | 135,767,393 | 135,767,393 | 6 | 2 |
| 9 | 135,767,465 | 135,775,026 | 6 | 2 |
| 9 | 135,775,055 | 135,784,905 | 6 | 2 |
| 9 | 135,785,304 | 135,785,332 | 6 | 2 |
| 9 | 135,785,463 | 135,788,272 | 6 | 2 |
| 9 | 135,788,899 | 135,914,352 | 6 | 2 |
| 11 | 3,193,928 | 3,256,058 | 4 | 0 |
| 11 | 3,257,511 | 3,363,846 | 4 | 0 |
| 11 | 3,364,167 | 3,621,635 | 4 | 0 |
| 11 | 44,014,589 | 44,077,272 | 4 | 0 |
| 12 | 150,442 | 402,160 | 7 | 3 |
| 12 | 405,941 | 1,478,570 | 5 | 1 |
| 12 | 1,483,235 | 1,829,016 | 5 | 1 |
| 12 | 1,830,943 | 1,841,245 | 5 | 1 |
| 12 | 1,843,214 | 1,850,384 | 5 | 1 |
| 12 | 1,851,156 | 1,869,709 | 5 | 1 |
| 12 | 1,872,687 | 1,878,392 | 5 | 1 |
| 12 | 1,879,385 | 4,441,843 | 5 | 1 |
| 12 | 11,512,492 | 11,543,406 | 4 | 0 |
| 14 | 102,098,960 | 102,098,960 | 4 | 0 |
| **Regions with gain in patients with partial response:** | | | |  |
| 2 | 95,327,887 | 103,834,965 | 0 | 3 |
| 2 | 103,838,000 | 104,208,418 | 0 | 3 |
| 2 | 104,209,064 | 115,535,914 | 0 | 3 |
| 2 | 115,536,169 | 115,545,975 | 0 | 3 |
| 2 | 115,546,549 | 124,146,759 | 0 | 3 |
| 2 | 124,147,407 | 127,240,551 | 0 | 3 |
| 3 | 122,529,408 | 122,621,112 | 0 | 3 |
| 3 | 122,630,062 | 124,308,719 | 0 | 3 |
| 3 | 138,797,007 | 138,877,031 | 0 | 3 |
| 5 | 168,187,391 | 170,179,881 | 0 | 3 |
| 6 | 101,646,801 | 109,026,266 | 0 | 3 |
| 6 | 109,518,400 | 118,387,544 | 0 | 3 |
| 6 | 118,656,959 | 118,720,581 | 0 | 3 |
| 6 | 118,720,704 | 118,875,931 | 0 | 3 |
| 6 | 118,876,092 | 122,804,112 | 0 | 3 |
| 6 | 122,804,194 | 124,371,998 | 0 | 3 |
| 6 | 124,372,841 | 124,461,689 | 0 | 4 |
| 6 | 124,470,365 | 134,198,817 | 0 | 3 |
| 6 | 134,199,014 | 144,455,364 | 0 | 3 |
| 6 | 150,159,464 | 150,394,817 | 0 | 3 |
| 6 | 150,397,495 | 150,642,796 | 0 | 3 |
| 6 | 150,644,384 | 154,722,801 | 0 | 3 |
| 20 | 14,761,461 | 14,782,742 | 3 | 6 |
| 20 | 15,931,765 | 16,168,007 | 4 | 7 |
| 20 | 16,168,311 | 16,838,230 | 4 | 7 |
| 20 | 19,344,295 | 21,561,885 | 4 | 7 |
| 20 | 21,562,155 | 21,568,222 | 4 | 7 |
| 20 | 26,177,105 | 26,305,579 | 5 | 8 |
| **Regions with loss in patients with stable disease:** | | |  |  |
| 1 | 603,590 | 721,290 | 6 | 2 |
| 1 | 723,317 | 3,680,729 | 6 | 2 |
| 1 | 3,681,831 | 5,188,954 | 6 | 2 |
| 1 | 10,563,671 | 10,580,516 | 7 | 3 |
| 1 | 10,580,540 | 10,596,530 | 7 | 3 |
| 1 | 10,596,713 | 16,630,945 | 7 | 3 |
| 1 | 17,235,698 | 17,589,005 | 8 | 4 |
| 1 | 17,589,048 | 19,773,433 | 8 | 4 |
| 1 | 19,775,492 | 23,238,023 | 8 | 4 |
| 1 | 23,264,359 | 23,268,849 | 9 | 5 |
| 1 | 23,271,432 | 23,408,483 | 9 | 5 |
| 1 | 23,409,538 | 23,449,418 | 9 | 5 |
| 1 | 23,449,635 | 25,583,291 | 9 | 5 |
| 1 | 25,583,341 | 25,583,341 | 9 | 5 |
| 1 | 29,633,708 | 29,669,796 | 9 | 5 |
| 17 | 27,492,042 | 27,492,042 | 5 | 1 |
| 22 | 17,057,138 | 17,510,720 | 5 | 1 |
| 22 | 18,235,288 | 22,263,914 | 5 | 1 |
| 22 | 22,266,808 | 22,294,301 | 5 | 1 |
| 22 | 22,583,115 | 22,999,206 | 5 | 1 |
| 22 | 23,002,377 | 23,300,887 | 5 | 1 |
| 22 | 23,301,565 | 26,975,215 | 5 | 1 |
| 22 | 26,979,848 | 26,991,234 | 5 | 1 |
| 22 | 26,992,055 | 28,423,844 | 5 | 0 |
| 22 | 28,426,022 | 28,472,291 | 5 | 0 |
| 22 | 28,475,176 | 28,502,440 | 5 | 0 |
| 22 | 28,502,815 | 28,502,815 | 5 | 0 |
| 22 | 28,502,867 | 28,537,115 | 5 | 1 |
| 22 | 28,537,992 | 28,537,992 | 5 | 1 |
| 22 | 28,538,878 | 28,540,492 | 5 | 1 |
| 22 | 28,541,025 | 29,041,332 | 5 | 1 |
| 22 | 29,041,877 | 29,046,240 | 5 | 1 |
| 22 | 29,052,951 | 29,076,148 | 5 | 1 |
| 22 | 29,667,606 | 29,668,004 | 5 | 1 |
| 22 | 29,668,199 | 32,475,303 | 5 | 1 |
| 22 | 32,476,600 | 32,936,813 | 5 | 0 |
| 22 | 32,937,108 | 36,116,080 | 5 | 0 |
| 22 | 36,117,854 | 36,129,851 | 5 | 0 |
| 22 | 36,135,349 | 36,868,791 | 4 | 0 |
| 22 | 36,872,750 | 37,376,404 | 5 | 0 |
| 22 | 37,377,737 | 37,380,380 | 6 | 0 |
| 22 | 37,382,144 | 40,465,263 | 6 | 0 |
| 22 | 40,465,803 | 42,682,557 | 6 | 1 |
| 22 | 42,682,786 | 43,530,982 | 6 | 1 |
| 22 | 43,531,129 | 43,536,126 | 6 | 1 |
| 22 | 43,542,719 | 43,542,719 | 6 | 0 |
| 22 | 43,544,823 | 43,558,487 | 6 | 0 |
| 22 | 43,558,926 | 45,940,934 | 6 | 0 |
| 22 | 45,943,203 | 46,665,006 | 5 | 0 |
| 22 | 46,665,670 | 51,234,455 | 5 | 0 |
| **Regions with loss in patients with partial response:** | | | |  |
| 3 | 59,957,830 | 59,957,830 | 2 | 6 |
| 3 | 59,958,060 | 60,001,874 | 2 | 6 |
| 3 | 60,002,455 | 60,059,959 | 2 | 6 |
| 4 | 91,505,240 | 91,505,240 | 4 | 8 |
| 4 | 91,507,015 | 91,574,079 | 4 | 8 |
| 4 | 91,577,127 | 91,577,127 | 4 | 8 |
| 4 | 92,066,971 | 92,425,224 | 4 | 8 |
| 4 | 178,969,298 | 178,969,724 | 3 | 7 |
| 4 | 178,973,203 | 179,775,385 | 3 | 7 |
| 4 | 179,786,141 | 179,799,574 | 3 | 7 |
| 4 | 180,696,728 | 181,103,729 | 3 | 7 |
| 4 | 181,105,111 | 181,892,770 | 3 | 8 |
| 4 | 181,894,845 | 182,810,934 | 3 | 7 |
| 4 | 182,814,197 | 182,966,224 | 3 | 8 |
| 4 | 182,967,545 | 184,177,384 | 4 | 8 |
| 4 | 184,180,384 | 184,180,384 | 4 | 8 |
| 4 | 188,095,501 | 191,027,923 | 3 | 7 |
| 8 | 42,639,312 | 43,353,486 | 3 | 7 |
| 8 | 43,360,692 | 43,824,048 | 3 | 7 |
| 9 | 6,012,734 | 6,168,335 | 0 | 4 |
| 9 | 6,169,135 | 6,268,922 | 0 | 5 |
| 9 | 6,270,908 | 6,899,149 | 1 | 5 |
| 9 | 6,969,634 | 7,305,031 | 0 | 4 |
| 9 | 7,306,383 | 8,258,659 | 0 | 4 |
| 9 | 8,258,921 | 14,785,224 | 0 | 4 |
| 9 | 14,785,294 | 14,849,045 | 0 | 4 |
| 9 | 15,033,952 | 15,739,131 | 1 | 5 |
| 9 | 15,742,241 | 19,060,937 | 0 | 4 |
| 9 | 19,064,604 | 19,315,386 | 0 | 5 |
| 9 | 19,315,923 | 20,860,495 | 0 | 4 |
| 9 | 20,868,287 | 20,917,493 | 0 | 5 |
| 9 | 20,920,713 | 26,241,963 | 0 | 4 |
| 9 | 26,435,047 | 30,329,758 | 0 | 4 |
| 9 | 30,330,466 | 31,929,553 | 0 | 4 |
| 9 | 31,930,138 | 31,972,254 | 0 | 4 |
| 9 | 31,972,671 | 32,175,970 | 0 | 4 |
| 9 | 32,179,169 | 36,105,264 | 0 | 4 |
| 9 | 36,110,749 | 39,110,886 | 0 | 4 |
| 12 | 33,039,426 | 33,039,426 | 0 | 4 |
| 12 | 67,815,985 | 67,878,134 | 0 | 4 |
| 12 | 88,575,652 | 88,580,274 | 0 | 4 |
| 12 | 88,588,208 | 89,719,376 | 0 | 4 |
| 12 | 89,724,927 | 89,743,394 | 0 | 4 |
| 12 | 93,151,544 | 93,160,220 | 0 | 4 |
| 12 | 97,156,037 | 97,182,999 | 0 | 4 |
| 12 | 99,286,696 | 99,727,131 | 0 | 4 |
| 14 | 20,425,911 | 21,298,404 | 1 | 5 |
| 14 | 21,298,988 | 23,396,734 | 1 | 5 |
| 14 | 23,397,720 | 23,468,457 | 1 | 5 |
| 14 | 23,468,912 | 23,473,410 | 1 | 5 |
| 14 | 23,473,706 | 23,725,239 | 1 | 5 |
| 14 | 31,254,386 | 31,316,919 | 1 | 5 |
| 14 | 32,347,353 | 32,364,733 | 1 | 5 |
| 14 | 34,840,772 | 34,847,229 | 2 | 6 |
| 14 | 34,849,920 | 34,851,996 | 2 | 6 |
| 14 | 36,387,728 | 36,387,874 | 1 | 6 |
| 14 | 36,389,629 | 36,467,230 | 1 | 5 |
| 14 | 49,946,421 | 51,220,624 | 1 | 5 |
| 14 | 73,372,933 | 74,634,084 | 1 | 5 |
| 16 | 6,761,165 | 6,763,141 | 1 | 5 |
| 16 | 6,763,216 | 6,780,301 | 1 | 5 |
| 16 | 6,781,830 | 6,790,673 | 1 | 5 |
| 16 | 6,791,533 | 6,802,327 | 1 | 5 |
| 17 | 5,359,190 | 5,519,516 | 4 | 8 |

# Table E. Genomic regions with high frequency of gain in patients with stable disease and concomitant loss in patients with partial response after pre-surgical chemotherapy

| **Chromosome** | **Start position** | **End position** | **Gain^a^** | | | **Loss^a^** | | **No. of genes** |
| --- | --- | --- | --- | --- | --- | --- | --- | --- |
|  |  |  | **Patients with stable disease (n = 9)** | | **Patients with partial response (n = 10)** | **Patients with stable disease (n = 9)** | **Patients with partial response (n = 10)** |  |
| **Regions with gain in patients with stable disease and loss in patients with partial response:** | | | | | |  |  |  |
| 9 | 46,587 | 4,163,335 | | 5 | 0 | 0 | 3 | 22 |
| 9 | 4,163,463 | 6,012,102 | | 5 | 0 | 0 | 3 | 31 |
| 9 | 6,012,734 | 6,168,335 | | 5 | 0 | 0 | 4 | 1 |
| 9 | 6,270,908 | 6,899,149 | | 5 | 0 | 1 | 5 | 9 |
| 9 | 6,900,898 | 6,965,101 | | 5 | 0 | 1 | 4 | 1 |
| 9 | 6,965,750 | 6,969,585 | | 5 | 0 | 1 | 4 | 1 |
| 9 | 6,969,634 | 7,305,031 | | 5 | 0 | 0 | 4 | 1 |
| 9 | 7,306,383 | 8,258,659 | | 5 | 0 | 0 | 4 | 2 |
| 9 | 8,258,921 | 14,785,224 | | 5 | 0 | 0 | 4 | 17 |
| 9 | 14,785,294 | 14,849,045 | | 5 | 0 | 0 | 4 | 1 |
| 9 | 14,850,060 | 15,032,733 | | 5 | 0 | 1 | 4 | 3 |
| 9 | 15,033,952 | 15,739,131 | | 5 | 0 | 1 | 5 | 11 |
| 9 | 15,742,241 | 19,060,937 | | 5 | 0 | 0 | 4 | 14 |
| 9 | 19,064,604 | 19,315,386 | | 5 | 0 | 0 | 5 | 5 |
| 9 | 19,315,923 | 20,860,495 | | 5 | 0 | 0 | 4 | 11 |
| 9 | 20,868,287 | 20,917,493 | | 5 | 0 | 0 | 5 | 1 |
| 9 | 20,920,713 | 26,241,963 | | 5 | 0 | 0 | 4 | 49 |
| 9 | 26,242,038 | 26,248,563 | | 5 | 0 | 1 | 4 | 0 |
| 9 | 26,256,181 | 26,263,018 | | 5 | 0 | 1 | 4 | 0 |
| 9 | 26,265,534 | 26,434,494 | | 5 | 0 | 1 | 4 | 0 |
| 9 | 26,435,047 | 30,329,758 | | 5 | 0 | 0 | 4 | 16 |
| 9 | 30,330,466 | 31,929,553 | | 5 | 0 | 0 | 4 | 8 |
| 9 | 31,930,138 | 31,972,254 | | 5 | 0 | 0 | 4 | 0 |
| 9 | 31,972,671 | 32,175,970 | | 5 | 0 | 0 | 4 | 0 |
| 9 | 32,179,169 | 36,105,264 | | 5 | 0 | 0 | 4 | 120 |
| 9 | 36,110,749 | 39,110,886 | | 5 | 0 | 0 | 4 | 41 |
| 9 | 86,639,109 | 100,380,227 | | 5 | 0 | 1 | 3 | 135 |
| 9 | 101,194,716 | 101,525,021 | | 5 | 0 | 1 | 3 | 3 |
| 12 | 150,442 | 402,160 | | 7 | 3 | 0 | 3 | 4 |
| 12 | 1,851,156 | 1,869,709 | | 5 | 1 | 0 | 3 | 2 |
| 12 | 33,039,426 | 33,039,426 | | 3 | 0 | 0 | 4 | 1 |
| 14 | 21,298,988 | 23,396,734 | | 3 | 0 | 1 | 5 | 175 |
| 14 | 23,397,720 | 23,468,457 | | 3 | 0 | 1 | 5 | 5 |
| 14 | 23,468,912 | 23,473,410 | | 3 | 0 | 1 | 5 | 1 |
| 14 | 23,473,706 | 23,725,239 | | 3 | 0 | 1 | 5 | 10 |
| 14 | 31,254,386 | 31,316,919 | | 3 | 0 | 1 | 5 | 0 |
| 14 | 32,347,353 | 32,364,733 | | 3 | 0 | 1 | 5 | 0 |
| 14 | 34,840,772 | 34,847,229 | | 3 | 0 | 2 | 6 | 1 |
| 14 | 34,849,920 | 34,851,996 | | 3 | 0 | 2 | 6 | 1 |
| 14 | 36,387,728 | 36,387,874 | | 2 | 0 | 1 | 6 | 1 |
| 14 | 49,946,421 | 51,220,624 | | 3 | 0 | 1 | 5 | 35 |
| 14 | 73,372,933 | 74,634,084 | | 3 | 0 | 1 | 5 | 30 |
| 14 | 102,098,960 | 102,098,960 | | 4 | 0 | 1 | 4 | 0 |
| **Regions with gain in patients with partial response and loss in patients with stable disease:** | | | | | |  |  |  |
| 1 | 3,681,831 | 5,188,954 | | 1 | 2 | 6 | 2 | 8 |
| 1 | 10,563,671 | 10,580,516 | | 0 | 1 | 7 | 3 | 1 |
| 1 | 10,580,540 | 10,596,530 | | 0 | 1 | 7 | 3 | 1 |
| 1 | 10,596,713 | 16,630,945 | | 0 | 1 | 7 | 3 | 116 |
| 1 | 29,633,708 | 29,669,796 | | 0 | 1 | 9 | 5 | 1 |
| 2 | 31,879,744 | 31,892,342 | | 0 | 2 | 3 | 0 | 0 |
| 6 | 124,372,841 | 124,461,689 | | 0 | 4 | 2 | 1 | 1 |
| 17 | 27,492,042 | 27,492,042 | | 1 | 2 | 5 | 1 | 1 |
| 17 | 30,807,348 | 31,281,886 | | 0 | 2 | 5 | 2 | 4 |
| 17 | 31,284,664 | 31,305,967 | | 0 | 2 | 5 | 2 | 1 |
| 17 | 31,306,768 | 31,334,371 | | 0 | 2 | 5 | 2 | 1 |
| 17 | 68,494,308 | 68,604,635 | | 0 | 2 | 5 | 2 | 0 |
| 17 | 70,575,888 | 72,130,512 | | 0 | 2 | 5 | 2 | 13 |
| 17 | 72,131,052 | 72,940,291 | | 0 | 2 | 5 | 2 | 27 |
| 17 | 75,338,503 | 75,345,606 | | 0 | 2 | 5 | 2 | 1 |
| 17 | 75,345,797 | 76,267,069 | | 0 | 2 | 5 | 2 | 17 |
| 17 | 76,268,506 | 76,885,117 | | 0 | 2 | 5 | 2 | 11 |
| 22 | 16,052,528 |  | | 0 | 1 | 5 | 1 | 4 |
| 22 | 17,057,138 | 17,510,720 | | 0 | 1 | 5 | 1 | 20 |
| 22 | 18,235,288 | 22,263,914 | | 0 | 1 | 5 | 1 | 115 |
| 22 | 22,583,115 | 22,999,206 | | 0 | 1 | 5 | 1 | 31 |
| 22 | 23,301,565 | 26,975,215 | | 0 | 1 | 5 | 1 | 73 |
| 22 | 26,979,848 | 26,991,234 | | 0 | 1 | 5 | 1 | 1 |
| 22 | 26,992,055 | 28,423,844 | | 0 | 1 | 5 | 0 | 12 |
| 22 | 28,426,022 | 28,472,291 | | 0 | 1 | 5 | 0 | 2 |
| 22 | 28,475,176 | 28,502,440 | | 0 | 1 | 5 | 0 | 1 |
| 22 | 28,502,815 | 28,502,815 | | 0 | 1 | 5 | 0 | 1 |
| 22 | 28,502,867 | 28,537,115 | | 0 | 1 | 5 | 1 | 1 |
| 22 | 28,537,992 | 28,537,992 | | 0 | 1 | 5 | 1 | 1 |
| 22 | 28,538,878 | 28,540,492 | | 0 | 1 | 5 | 1 | 1 |
| 22 | 28,541,025 | 29,041,332 | | 0 | 1 | 5 | 1 | 2 |
| 22 | 29,668,199 | 32,475,303 | | 0 | 1 | 5 | 1 | 72 |
| 22 | 32,476,600 | 32,936,813 | | 0 | 1 | 5 | 0 | 16 |
| 22 | 32,937,108 | 36,116,080 | | 0 | 1 | 5 | 0 | 20 |
| 22 | 36,117,854 | 36,129,851 | | 1 | 1 | 5 | 0 | 1 |
| 22 | 36,872,750 | 37,376,404 | | 0 | 1 | 5 | 0 | 9 |
| 22 | 37,377,737 | 37,380,380 | | 0 | 1 | 6 | 0 | 0 |
| 22 | 37,382,144 | 40,465,263 | | 0 | 1 | 6 | 0 | 87 |
| 22 | 40,465,803 | 42,682,557 | | 0 | 1 | 6 | 1 | 61 |
| 22 | 42,682,786 | 43,530,982 | | 0 | 1 | 6 | 1 | 20 |
| 22 | 43,531,129 | 43,536,126 | | 0 | 1 | 6 | 1 | 1 |
| 22 | 43,542,719 | 43,542,719 | | 0 | 1 | 6 | 0 | 0 |
| 22 | 43,544,823 | 43,558,487 | | 0 | 1 | 6 | 0 | 1 |
| 22 | 43,558,926 | 45,940,934 | | 0 | 1 | 6 | 0 | 32 |
| 22 | 45,943,203 | 46,665,006 | | 0 | 1 | 5 | 0 | 15 |
| 22 | 46,665,670 | 51,234,455 | | 0 | 1 | 5 | 0 | 55 |

^a^Number of patients with copy number aberration in at least one metastasis
